# Exploring the Bioactive Secondary Metabolites of Two Argentine *Trichoderma afroharzianum* Strains

**DOI:** 10.3390/jof11060457

**Published:** 2025-06-17

**Authors:** Rodrigo José Nunes Calumby, Antonella Santone, Estefanía Butassi, Laura Andrea Svetaz, Márcia de Souza Carvalho Melhem, Sebastián Pablo Rius, Valeria Alina Campos-Bermudez

**Affiliations:** 1 Centro de Estudios Fotosintéticos y Bioquímicos (CEFOBI), Consejo Nacional de Investigaciones Científicas y Técnicas (CONICET), Universidad Nacional de Rosario, Suipacha 531, Rosario S2002LRK, Santa Fe, Argentina; nunescalumby@cefobi-conicet.gov.ar (R.J.N.C.); santone@cefobi-conicet.gov.ar (A.S.); rius@cefobi-conicet.gov.ar (S.P.R.); 2 Área Farmacognosia, Facultad de Ciencias Bioquímicas y Farmacéuticas, Universidad Nacional de Rosario, Suipacha 531, Rosario S2002LRK, Santa Fe, Argentina; estefaniabutassi@gmail.com (E.B.); svetaz@rosario-conicet.gov.ar (L.A.S.); 3 Programa de Pós-Graduação em Doenças Infecciosas e Parasitárias, Universidade Federal do Mato Grosso do Sul, Campo Grande 79070-900, MS, Brazil; melhemmr@uol.com.br; 4 Laboratório de Investigação Médica-LIM53, Hospital das Clínicas, Faculdade de Medicina, Universidade de São Paulo, São Paulo 01246-903, SP, Brazil; 5 Programa de Pós-Graduação em Doenças Tropicais, Faculdade de Medicina, Universidade Estadual Paulista—UNESP, Campus Botucatu, São Paulo 01049-010, SP, Brazil

**Keywords:** *Trichoderma*, bioactive compounds, antimicrobial, antibiofilm, antioxidant, HPLC/MS analysis

## Abstract

*Trichoderma* spp. produce diverse secondary metabolites with biological activity. This study explored the antimicrobial, antibiofilm, antioxidant, and cytotoxic properties of metabolites from two native *Trichoderma* strains, 10BR1 and UEPA AR12, isolated from rhizospheric soils. Organic extracts from both strains demonstrated broad-spectrum antimicrobial activity, inhibiting Gram-positive and Gram-negative bacteria, as well as various *Candida* species, with notable efficacy against *Staphylococcus aureus* (MICs: 15.6–31.25 µg/mL). The extracts also showed antibiofilm activity, with UEPA AR12 exhibiting the highest inhibition against *Escherichia coli* (81.8%), *Enterococcus faecalis* (92.8%), *Candida albicans* (87.9%), and *Candida parapsilosis* (89.3%). Antioxidant activity, assessed via DPPH assay, revealed a dose-dependent radical scavenging effect (12.88% to 39.67% at 7.8–1000 µg/mL). Cytotoxicity assays indicated that UEPA AR12 extracts were more cytotoxic (IC_50_: 202.5–234.3 µg/mL) than 10BR1 (IC_50_: 368.7–602.1 µg/mL) in non-tumor cells, with similar trends in tumor cells (Huh7). HPLC/MS analysis identified 21 metabolites in the extracts. Genomic analyses, supported by *rpb2* gene and phylogenetic clustering, confirmed that both strains were *T. afroharzianum*. FUNGISMASH revealed multiple biosynthetic gene clusters, predominantly Type I polyketide synthase (T1PKS). Additionally, targeted genomic analyses did not detect mycotoxin-related genes. These findings highlight the antimicrobial, antibiofilm, and antioxidant potentials of these strains, positioning them as sources of bioactive metabolites for pharmaceutical applications.

## 1. Introduction

Antimicrobial resistance is one of the most critical public health threats worldwide, driven by the excessive and improper use of antibiotics. This phenomenon has accelerated the emergence of multidrug-resistant pathogens, undermining the efficacy of conventional therapies and increasing morbidity and mortality rates [1,2,3]. The challenge is further exacerbated by the formation of microbial biofilms, which create protective matrices that reduce drug penetration and protect pathogens from both antimicrobials and host immune responses [4]. Due to the scarcity of new antibiotics in recent decades, there is an urgent need to explore alternative strategies, including the use of natural products capable of inhibiting pathogen growth and biofilm formation [1].

Common human pathogens, such as *Staphylococcus aureus* [5], *Enterococcus faecalis* [6], *Escherichia coli* [7], and *Candida* species [8], are particularly concerning due to their increasing resistance to multiple antibiotics. These microorganisms have developed mechanisms such as the acquisition of resistance genes (e.g., *mecA* in *S. aureus*, *vanA* in *E. faecalis*), production of antibiotic-degrading enzymes, and overexpression of efflux pumps (e.g., NorA in *S. aureus*, AcrAB-TolC in *E. coli*) [5,6,7], all of which significantly compromise treatment efficacy. Moreover, their ability to form biofilms on both biotic and abiotic surfaces further reduces antimicrobial penetration and shields cells from host immune responses [9]. Consequently, infections caused by these pathogens often become chronic and recurrent, underscoring the need for novel therapeutic solutions [10].

To overcome these limitations, natural products, particularly those derived from microorganisms, remain a promising source of structurally diverse bioactive compounds. Filamentous fungi from soil ecosystems have gained attention as reservoirs of such metabolites, including those with antimicrobial, antioxidant, and cytotoxic activities [11,12,13,14]. These fungi produce a wide range of secondary metabolites, such as alkaloids, flavonoids, phenols, steroids, and terpenoids [15], that play key roles in ecological interactions and show potential for pharmaceutical development.

Among these fungi, the genus *Trichoderma* is especially notable. Known for its versatility and adaptability, *Trichoderma* species have been extensively studied in agriculture for their roles in plant growth promotion, soil health improvement, and biocontrol of phytopathogens [16,17]. These beneficial properties are largely attributed to the production of secondary metabolites and hydrolytic enzymes [18]. Importantly, several of these metabolites have demonstrated activities relevant to human health, including antibacterial, antifungal, antioxidant, anti-inflammatory, and anti-cancer effects [19].

While the biotechnological potential of several *Trichoderma* species has been extensively studied, particularly in agricultural contexts, *T. afroharzianum* stands out due to its growing use as a biocontrol agent and plant growth promoter across diverse crops [20,21]. This species, part of the *T. harzianum* sensu lato complex, is known for its strong root colonization capacity, adaptability to variable environmental conditions, and ability to produce bioactive metabolites [22]. Despite its increasing agricultural relevance, its potential as a source of pharmacologically active secondary metabolites remains largely unexplored. In contrast to other *Trichoderma* species, the specific compounds produced by *T. afroharzianum*, the diversity of its biosynthetic pathways, and the biological activities of its metabolites are still poorly characterized.

To address these knowledge gaps, we investigated the antimicrobial, antibiofilm, antioxidant, and cytotoxic activities of metabolites produced by two native *T. afroharzianum* strains isolated from Argentine soil. We further characterized these metabolites using HPLC/MS and employed genome mining through FUNGISMASH to identify biosynthetic gene clusters involved in secondary metabolism. This integrated approach expands the current understanding of *T. afroharzianum*’s metabolic potential and supports its evaluation as a source of novel compounds for future therapeutic applications.

## 2. Materials and Methods

### 2.1. Chemical Materials

The chemicals used were obtained from their respective suppliers, including ethyl acetate and methanol (Dorwill, Buenos Aires, Argentina); dimethyl sulfoxide (DMSO) and crystal violet (Biopack, Buenos Aires, Argentina); 1,1-diphenyl-2-picrylhydrazyl (DPPH), 3-(4,5-dimethyl-2-thiazolyl)-2,5-diphenyl-2H-tetrazolium bromide (MTT), and morpholinepropanesulfonic acid (MOPS) (Sigma-Aldrich, St. Louis, MO, USA); and fetal bovine serum (FBS) (Gibco, Thermo Fisher Scientific, Waltham, MA, USA).

### 2.2. Fungal Strains

This study used two strains of *T. afroharzianum*, 10BR1 (CCC 115-23) and UEPA AR12 (CCC 175-23), which are part of the collection of the Plant–Microorganism Interaction Physiology Laboratory of the Center for Photosynthetic and Biochemical Studies (CEFOBI) and were maintained at the Mycological Reference Center (CEREMIC) of the Faculty of Biochemical and Pharmaceutical Sciences of the National University of Rosario. Both strains were isolated from soil samples collected in the southern part of the Santa Fe province, in the Pampas region of Argentina (32°25′00″ S, 61°54′00″ W), with sampling permit No. 255/2018, file 02101-0019157-3.

For isolation, 14 soil samples were collected. From each sample, four 10 g subsamples were taken and suspended in 100 mL of sterile distilled water. The mixtures were stirred for 30 min and allowed to settle for 10 min to enable decantation. Serial dilutions (1:10, 1:100, and 1:1000) were then prepared, and aliquots of 0.1 mL from the original and diluted suspensions were plated in triplicate on *Trichoderma harzianum* Selective Agar Base (HiMedia Laboratories, Mumbai, India). Plates were incubated at 28 °C on a 12 h light/12 h dark cycle for 7 to 10 days. Colonies with rapid growth, abundant sporulation, and green-to-yellow-green pigmentation, typical of the genus *Trichoderma*, were selected. Part of the mycelia from these colonies was transferred to fresh Potato Dextrose Agar (PDA) medium (Difco, Becton Dickinson, Sparks, MD, USA) and incubated under the same conditions for 7 days. The conidia were then harvested and stored in 20% glycerol at −80 °C.

We selected strains 10BR1 and UEPA AR12 from an initial screening of 25 *Trichoderma* isolates. All strains were subjected to a series of in vitro assays to evaluate their potential for plant growth promotion (PGP) and biocontrol activity. For PGP assessment, we measured indole-3-acetic acid (IAA) production using the Salkowski reagent [23], phosphate solubilization [24], and cellulase secretion [25]. Biocontrol potential was evaluated through co-cultivation assays against a panel of phytopathogenic fungi, including *Diaporthe caulivora*, *Macrophomina phaseolina*, *Fusarium oxysporum*, *Fusarium verticillioides*, and *Botrytis cinerea*. Based on their superior performance across multiple assays, strains 10BR1 and UEPA AR12 were selected for further genomic and functional characterization [26].

### 2.3. Identification of Trichoderma

#### 2.3.1. Phenotypic Characterization

Fungal cultures preserved in glycerol (−80 °C) were reactivated by culturing on Petri dishes with PDA medium. The plates were incubated at 28 °C for approximately 10 days. At the end of the incubation period, the macroscopic and microscopic characteristics of the colonies were evaluated and compared with those described in the literature to confirm their classification within the genus *Trichoderma*. Macroscopic characteristics evaluated included colony color, texture, size, and pigmentation [27,28,29].

Microscopic characteristics were assessed using microcultures prepared on lactrimel agar using the Ridell technique [28]. Observations were made using a bright-field microscope with 10× and 40× objectives to analyze the fungal structural details.

#### 2.3.2. Genome Sequences and Species Identification

To confirm the taxonomic classification of *Trichoderma* strains, cultures were grown in Potato Dextrose Broth (PDB) liquid medium (Difco, Becton Dickinson, Sparks, MD, USA), and mycelia were collected for DNA extraction and purification using the NucleoSpin Soil kit (Macherey Nagel^®^, Düren, Germany). The fungal DNAs were then sent to Macrogen Inc. (Seoul, Republic of Korea) for complete sequencing. For species identification, the sequences obtained were compared with those available in public databases (GenBank DNA sequence database, National Center for Biotechnology Information BLAST). The complete genomic sequences of strains 10BR1 and UEPA AR12 of *Trichoderma* were deposited in GenBank under accession numbers JBDIXD000000000 and JBDIXE000000000, respectively.

#### 2.3.3. Phylogenetic and Taxonomic Characterization of *Trichoderma* Strains

For accurate taxonomic identification of strains 10BR1 and UEPA AR12, the *rpb2* gene, widely used for species delimitation in *Trichoderma*, was retrieved from the whole-genome assemblies. Sequence identity was determined by BLASTn [30] using publicly available *Trichoderma* sequences.

To further elucidate the phylogenetic relationships of strains 10BR1 and UEPA AR12 with other *Trichoderma* species, phylogenetic analyses were performed using MEGA11 software (v11.0.13) [31]. Sequences of *rpb2* gene were aligned using ClustalW (v2.1) [32], and evolutionary distances were estimated. A phylogenetic tree was constructed using the neighbor-joining algorithm with 1000 bootstrap replicates to assess the robustness of the inferred clades.

### 2.4. Extraction of Secondary Metabolites

To prepare the metabolite extracts, each *T. afroharzianum* strain was cultured in Petri dishes with PDA medium and grown at 28 °C in a microbiological incubator for 10 days. Conidia were collected in 2 mL of sterile water using a Drigalsky spatula (Biologix Group Limited, Jinan, China) and counted in a Neubauer chamber. A 1 mL aliquot of the conidial suspension (10^6^ conidia/mL) from each fungal strain was inoculated into 200 mL of PDB. Growth was performed at 28 °C with shaking (180 rpm) for 30 days.

After incubation, the culture was filtered through a vacuum system to separate the mycelium from the supernatant. The resulting supernatants were subjected to liquid–liquid partitioning with ethyl acetate (three times) in a 1:1 ratio. After extraction, the extracts were filtered and transferred to a rotary evaporator under reduced pressure (0.09 MPa) at 40 °C to obtain the crude extracts. For further studies, the dried extracts were dissolved in DMSO for microbiological and cytotoxicity assays, and in methanol for HPLC analysis.

### 2.5. Antimicrobial Activity of Extracts

#### 2.5.1. Microorganisms

Strains from the American Type Culture Collection (ATCC) (Rockville, MD, USA) were used for antimicrobial evaluation. The selected bacteria were gram-positive *E. faecalis* (ATCC 29212) and *S. aureus* (ATCC 25923), and gram-negative *Salmonella enterica* serovar Typhimurium (ATCC 14028s) and *E. coli* (ATCC 25922 and ATCC 35218). Regarding the studied fungi, five species of *Candida* were considered: *C. albicans* (ATCC 10231), *C. parapsilosis* (ATCC 22019), *C. tropicalis* (ATCC 200956), *C. krusei* (ATCC 6258), and *C. glabrata* (ATCC 2950). In addition, two clinical isolates of *C. albicans* (CCC 132-15 and CCC 191-13) from CEREMIC were included.

#### 2.5.2. Determination of the Minimum Inhibitory Concentration

The Minimum Inhibitory Concentration (MIC) of each metabolite extract was determined using the broth microdilution technique in 96-well microplates with covers and flat bottoms (Greiner BioOne, Wemmel, Belgium), according to the guidelines of the Clinical and Laboratory Standards Institute [33,34]. MIC values were determined in Mueller Hinton Broth medium (Britania, Buenos Aires, Argentina) for bacteria and in RPMI-1640 medium with 2% *w*/*v* glucose (Sigma-Aldrich, St. Louis, MO, USA) buffered at pH 7.0 with MOPS for *Candida* strains.

A stock solution of the dried crude extracts was prepared at 50 mg/mL in 100% DMSO. A working solution in 2000 μg/mL was then obtained by diluting the stock with the appropriate culture medium. This working solution was serially diluted in the same medium directly in the microplate to yield final concentrations ranging from 1000 to 7.8 μg/mL. The DMSO concentration in all wells was kept at or below 1%. Finally, the microbial inoculum was added.

The inoculum suspension was prepared in sterile saline solution (0.85% *w*/*v*) and adjusted to a turbidity equivalent to the 0.5 McFarland standard by measuring the optical density at 625 nm (OD625) using a spectrophotometer, corresponding to an approximate bacterial concentration of 1.5 × 10^8^ CFU/mL and 1–5 × 10^6^ CFU/mL for yeast cells. For bacteria, the resulting suspension was vortexed and diluted at a 1:10 ratio to achieve a concentration of 10^7^ CFU/mL. Then, 5 µL of the inoculum were added to each microplate well, resulting in a final concentration of 5 × 10^5^ CFU/mL. For *Candida* strains, the resulting suspension was diluted at a ratio of 1:50, followed by 1:20, to achieve a final fungal cell concentration of 1–5 × 10^3^ CFU/mL. Then, 100 μL of the inoculum were added to each well, followed by 100 μL of the antifungal compound solution, resulting in a final cell concentration of 0.5–2.5 × 10^3^ CFU/mL.

Wells were also designated for growth control (GC, culture medium with inoculum but without the extract), sterility control (SC, culture medium without extract and with sterile distilled water instead of inoculum), and extract control (EC, culture medium with the extract but with sterile distilled water instead of inoculum). Ampicillin and Amphotericin B were used as positive controls. Each test was performed in triplicate. The plates were incubated at 37 °C for 24 h in a microbiological incubator. To aid visualization, Triphenyl Tetrazolium Chloride (TTC) (Sigma-Aldrich, St. Louis, MO, USA), a microbial growth indicator, was used by adding 20 µL of 0.1% (*w*/*v*) solution and incubating for an additional 3 h in the dark. MIC was defined as the lowest concentration at which visible growth was inhibited, corresponding to MIC_100_, as it reflects the point at which no growth was detectable.

### 2.6. Antibiofilm Assay

#### 2.6.1. Biofilm Formation Inhibition in Bacteria

The antibiofilm activity of the extract was evaluated against a Gram-negative bacterium (*E. coli* ATCC 35218) and a Gram-positive bacterium (*E. faecalis* ATCC 29212), both of which were previously confirmed as biofilm formers. Starting from an overnight culture of these strains in LB broth at 37 °C for 24 h, a 1:10 dilution was prepared, and the optical density (OD) was measured at 660 nm. The cultures were adjusted to an initial OD of 0.05 and incubated for 72 h at 37 °C with a two-fold serial dilution of the extract (500 to 15.6 μg/mL). Controls included bacterial cultures without the extract, culture media supplemented with the tested extract concentrations, but without bacterial inoculation, and culture media without either the extract or inoculation. Gentamicin (4 µg/mL) was used as a positive control.

The assay was performed according to the protocol described by O’Toole et al. [35]. Briefly, planktonic cells were removed by washing thrice with sterile water. The adherent cells were fixed and stained with 0.1% (*w*/*v*) crystal violet for 10 min. After staining, the wells were washed three times with sterile water to remove excess stain and then allowed to air-dry. The crystal violet bound to the biofilm was solubilized using an ethanol:acetone solution (80:20), and the absorbance was measured at 580 nm using an Epoch 2 microplate reader (BioTek, Winooski, VT, USA), with the ethanol:acetone solution serving as the blank. Additionally, three wells per row were set aside for complete homogenization of the biofilm-adherent cells and total biomass quantification. The optical density at 660 nm was measured to estimate the total biomass. Biofilm formation was determined as the ratio between the optical density of crystal violet-stained biofilms and turbidity of growth (OD_580_/OD_660_).

#### 2.6.2. Inhibition of Biofilm Formation in *Candida*

The assay was conducted using the clinical isolates of *C. albicans* (CCC 173–22) and *C. parapsilosis* (CCC 124–2000) obtained from CEREMIC. The methodology was adapted from Pierce et al. [36], with some modifications. Both *Candida* strains were cultured in yeast extract, peptone, and dextrose (YPD) broth for 24 h on an orbital shaker at 30 °C. Planktonic cells were collected by centrifugation (3000× *g*, 5 min), washed twice with phosphate-buffered saline (PBS), and adjusted to a final concentration of 1 × 10^6^ CFU/mL in RPMI-1640 medium supplemented with L-glutamine, 1.8% glucose, and buffered with 0.165 M MOPS at pH 7.

The inhibition of biofilm formation was evaluated using 96-well polystyrene microplates. For this, 100 μL of yeast cell suspension were added to each well, along with 100 μL of the two extracts in a two-fold serial dilution (1000–15.6 µg/mL). The biofilm formation control consisted of 100 μL of culture medium without the sample. Amphotericin B (2 µg/mL) was used as a positive control. The plates were statically incubated for 48 h at 37 °C. After biofilm formation, the liquid content was discarded, and the wells were gently washed with sterile PBS.

The metabolic activity of biofilms was quantified using a modified tetrazolium reduction assay. Briefly, 100 μL of a pre-warmed MTT solution (0.5 mg/mL in PBS) were added to each well. The plates were incubated for 4 h at 37 °C in the dark. After incubation, the MTT solution was removed, and the wells were washed with PBS. DMSO was then added to solubilize formazan, the product of MTT reduction. Formazan formation was measured at 540 nm using a microplate reader. All measurements were performed in triplicate.

#### 2.6.3. Biofilm Formation Inhibition Percentage and IC_50_ Determination

The percentage of biofilm formation inhibition in the bacterial and *Candida* strains was calculated by comparing the absorbance (A) values of treated wells to those of control wells without extracts, using the formula: Inhibition (%) = [(A_control_ − A_treated_)/A_control_] × 100. Average values ± standard deviation (SD) were used to generate dose-response curves, plotting inhibition (%) against the concentration of *T. afroharzianum* extracts. Dose-response curves were constructed and analyzed using GraphPad Prism software (version 8.0), fitting the data to a nonlinear regression model (log[inhibitor] vs. response—variable slope).

The IC_50_ values for each extract were determined from the fitted curves. IC_50_ was defined as the extract concentration required to inhibit cell viability or biofilm formation by 50% relative to the untreated control. These values were calculated using the equation of the fitted trendline derived from the experimental data.

### 2.7. Antioxidant Activity Assay

The antioxidant activity of the extracts was evaluated using the DPPH free radical scavenging assay, following the method described by Mollaei et al. [37]. Extracts were dissolved in methanol, and 50 μL aliquots at various concentrations (1000, 500, 250, 125, 62.5, 31.2, 15.6, and 7.8 μg/mL) were mixed with 550 μL of a DPPH solution (0.05 mg/mL in methanol). A blank control containing only methanol was included for absorbance measurements. After incubation at room temperature for 30 min, absorbance was measured at 517 nm using a Jenway 6705 UV-Vis spectrophotometer. The percentage of radical scavenging activity was calculated according to the following equation:

Radical Scavenging Activity = (A_control_ − A_sample_/A_control_) × 100

### 2.8. Cytotoxicity Assays

#### 2.8.1. Non-Tumor Cells

Vero cells (ATCC CCL-81), derived from the kidney tissue of a normal adult African green monkey, HEK293 cells (ATCC CRL-1573), derived from the kidney of a human embryo, and HaCaT cells (immortalized human keratinocytes, CVCL 0038), were cultured in Dulbecco’s modified Eagle’s medium (DMEM) (Gibco, Thermo Fisher Scientific, Waltham, MA, USA) supplemented with 10% FBS (*w*/*v*), 100 U/mL Penicillin, and Streptomycin (Invitrogen, Carlsbad, CA, USA). The cell lines were purchased from ATCC, and their authenticity was confirmed by standard Short Tandem Repeat (STR) analysis. Cultures were maintained in a humidified incubator at 37 °C with 5% CO_2_ and periodically checked for mycoplasma contamination using 4,6-diamidino-2-phenylindole staining and PCR.

Cells were seeded in 96-well plates at a density of 7 × 10^3^ to 15 × 10^3^ cells per well, depending on the cell line, 24 h before treatment. Fungal extracts (100 mg/mL) were initially dissolved in medium containing 10% FBS at a concentration of 1000 µg/mL and then subjected to half serial dilutions in medium containing 10% FBS to obtain final concentrations for different treatments (500 µg/mL, 250 µg/mL, 125 µg/mL, 62.5 µg/mL, 31.25 µg/mL, 15.625 µg/mL, and 7.8125 µg/mL). As a control, cells were incubated with medium containing 10% FBS and 1% DMSO.

After 24 h of treatment, the cells were stained with MTT (0.5 mg/mL) for 4 h at 37 °C. After removing the culture medium, the formazan crystals were dissolved in DMSO, and the absorbance was measured at 570 nm using a microplate reader.

#### 2.8.2. Tumor Cells

The cytotoxic properties of *T. afroharzianum* extracts were also evaluated by the reduction of MTT by the mitochondrial dehydrogenase of viable Huh7 human hepatoma cells, which resulted in the production of a blue formazan product that could be quantified spectrophotometrically. Huh7 cells were seeded in 96-well plates at a density of 5000 cells per well. After 24 h of attachment, the cells were treated with different concentrations of the extracts (7.8 µg/mL to 1000 µg/mL) for 72 h. After treatment, MTT was added to the culture medium to assess the cell viability [38]. The absorbance of the formazan product, generated by mitochondrial activity of viable cells, was measured at 540 nm (reference filter 650 nm) using a DTX 880 multimode detector (Beckman Coulter Inc., Fullerton, CA, USA). The results are expressed as a percentage of the absorbance of the control cells.

### 2.9. HPLC/MS Analysis

To identify the compounds present in the supernatant extracts, the samples were dissolved in methanol and filtered prior to injection into the HPLC-MS system. The analysis was performed using an Ultimate 3000 RSLC liquid chromatograph (Dionex, Thermo Fisher Scientific, Waltham, MA, USA) coupled with a TSQ Quantum Access Max mass detector (Thermo Fisher Scientific, Waltham, MA, USA) and a triple quadrupole (QqQ) mass spectrometer.

The chromatographic conditions were based on a protocol adapted from Stracquadanio et al. [39]. The mobile phase consisted of Milli-Q water containing 0.1% formic acid (phase A) and acetonitrile containing 0.1% formic acid (phase B). The flow rate was maintained at 0.2 mL/min, and the separation was performed on a Hypersil GOLD C18 column (Thermo Fisher Scientific, Waltham, MA, USA) with dimensions of 50 mm × 2.1 mm and a particle size of 1.9 µm. The column temperature was set to 40 °C, and the autosampler was maintained at 20 °C. The total run time was approximately 40 min with an injection volume of 10 µL. The gradient program was as follows: 95% water and 5% acetonitrile (time 0 min), maintained for 5 min, then switched to 5% water and 95% acetonitrile for 35 min, and returned to 95% water and 5% acetonitrile between 37 and 38 min.

For mass spectrometry analysis, the parameters were optimized using a compound with a structure similar to that of the target metabolites. The ionization parameters included ESI positive/negative mode, with a spray voltage of 3000 V, vaporizer temperature of 200 °C, capillary temperature of 300 °C, sheath gas pressure of 35 AU, and auxiliary gas pressure of 2 AU. The tube–lens offset was set to 86.

### 2.10. Bioinformatic Analysis of Secondary Metabolite Biosynthesis Pathways

Bioinformatics analysis was conducted using the Fungal Secondary Metabolite Analysis SHell (FUNGISMASH) portal (https://fungismash.secondarymetabolites.org/ (accessed on 15 February 2025)), which identified genomic regions linked to secondary metabolite biosynthesis pathways. This approach provided detailed insights into the location of gene clusters, enzymes involved in metabolite synthesis, and types of compounds potentially produced, underscoring the biotechnological potential of *Trichoderma* strains 10BR1 and UEPA AR12.

### 2.11. Statistical Analysis

All statistical analyses were conducted using GraphPad Prism software (version 8.0). Data from antioxidant, biofilm inhibition, and cytotoxicity assays were expressed as mean ± standard deviation (SD) from at least three independent experiments. Comparisons between extracts at different concentrations and for each condition (DPPH assay, biofilm inhibition, and cytotoxicity in different cell lines) were performed using two-way ANOVA followed by Tukey’s post-hoc test, with statistical significance considered at *p* < 0.05.

Additionally, the selectivity index (SI), calculated as the ratio between the IC_50_ value and the MIC for each extract and pathogen, was used to evaluate the safety profiles of the extracts.

## 3. Results

A total of 25 *Trichoderma* strains were evaluated for antagonistic activity against phytopathogens (*D. caulivora*, *M. phaseolina*, *F. oxysporum*, *F. verticillioides*, *B. cinerea*), as well as for relevant biological traits, including IAA production, phosphate solubilization, and cellulase secretion. Based on this preliminary screening [26], two strains, 10BR1 and UEPA AR12, which exhibited the highest and most consistent antimicrobial activity, were selected for further chemical and biological characterization.

### 3.1. Morphological Identification and Molecular Analysis of T. afroharzianum

**Figure 1:**
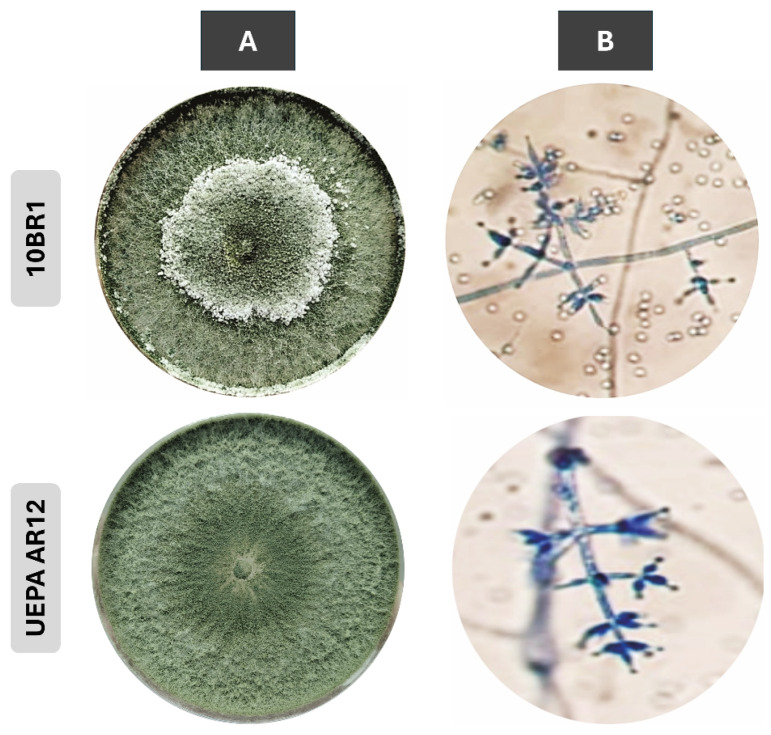
Morphological view of *T. afroharzianum* strains 10BR1 (CCC 115-23) and UEPA AR12 (CCC 175-23) on PDA (**A**) and microscopic observation of microcultures on lactrimel agar stained with cotton blue lactophenol at 400× magnification (**B**). Microscopic images illustrate general morphological features; scale bars are not included due to the qualitative nature of the observations and imaging conditions.

The two *Trichoderma* strains studied were native isolates obtained from the soil that interacted with plant roots in the rhizosphere. These strains were initially identified based on their colony morphology and microscopic characteristics (Figure 1). Colonies generally showed rapid growth, initially translucent white, which later developed sporulation tufts ranging from light green to opaque green, first at the edges and then spread throughout the entire colony. Microscopically, hyaline hyphae were observed together with pyramidal, branched conidiophores. The phialides were arranged in whorls and adopted a flask-like shape with sharp attenuation near the apex. The conidia were (sub-)spherical with smooth walls and pale green to subhyaline tones. Additionally, terminal and intercalary chlamydospores were observed, which were characterized by their hyaline appearance and smooth walls.

To further characterize the strains under investigation and gain insight into their species classification, we extracted genomic DNA from the isolates and sent the samples for sequencing. The obtained sequences were assembled and analyzed to identify the *rpb2* gene region. Strain 10BR1 shares 99.82% similarity with *T. afroharzianum* isolates Tr96 (accession number: OP374181.1), Tr84A (accession number: OP374178.1), and Tr129 (accession number: OP374183.1), all deposited in 2022 from Turkey, with an E-value of 0 and 100% coverage. In comparison, UEPA AR12 exhibited 100% similarity with *T. afroharzianum* strain Tri-1 (accession number: OP102132.1), isolated from soil in China, for biocontrol against *Sclerotinia sclerotiorum* in oilseed rape [40], with an E-value of 0 and 100% coverage (Supplementary Table S1).

**Figure 2:**
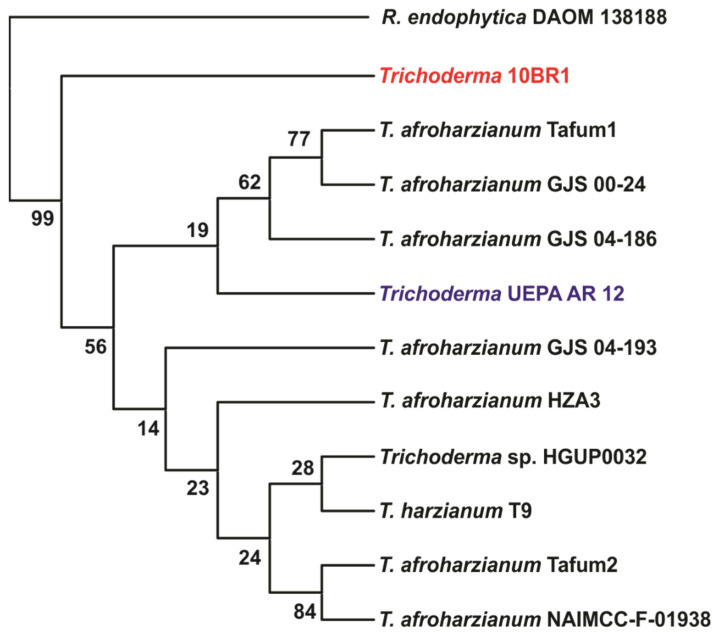
Phylogenetic tree based on the *rpb2* gene sequence of *T. afroharzianum* strains 10BR1 (CCC 115-23) and UEPA AR12 (CCC 175-23). For evolutionary inference of the strains, 1000 bootstrap trees were constructed using *rpb2* gene alignment and distance values inferred using MEGA11 (v11.0.13) software. Consensus was obtained from these trees using the neighbor-joining method. The investigated strains are highlighted in red (10BR1) and blue (UEPA AR12), with *Rhizoctonia endophytica* DAOM 138188 serving as the outgroup, forming the root of the tree.

Phylogenetic analysis, as shown in the consensus tree (Figure 2), revealed that strains UEPA AR12 and 10BR1 clustered with other *T. afroharzianum* isolates, forming a consistent group within the species. However, UEPA AR12 was more closely related to strains GJS 04-186, GJS 00-24, and Tafum1, while 10BR1 occupied a more distinct position, forming a unique subclade within the overall cluster. This distinct positioning may reflect the genetic variations that set 10BR1 apart from the others. The bootstrap values at the key nodes reinforce the reliability of the observed evolutionary relationships, although some relatively low values indicate the need for further analysis to confirm these associations. The complete phylogenetic tree, including additional *Trichoderma* species and all isolates used in this study, is shown in Supplementary Figure S1.

### 3.2. Antimicrobial Activity

The organic extracts obtained from the 30-day culture supernatant of *T. afroharzianum* strains 10BR1 (CSE10BR1) and UEPA AR12 (CSEAR12) exhibited broad-spectrum antimicrobial activity, effectively inhibiting both Gram-positive and Gram-negative bacteria, as well as several strains of *Candida*. The MICs of the extracts against the evaluated microorganisms are detailed in Table 1. It was observed that the extracts showed more pronounced antibacterial activity than antifungal activity. Specifically, they showed significant antibacterial activity against *S. aureus* (MICs of 15.6 and 31.25 µg/mL), moderate activity against *E. faecalis* (MICs of 125 and 250 µg/mL) and *S. Typhimurium* (MIC of 250 µg/mL), and low activity against *E. coli* (MIC of 500 µg/mL).

With regard to antifungal activity, the extracts showed moderate activity against *C. krusei* (MICs of 250 and 500 µg/mL) and low activity against *C. tropicalis* (MICs of 500 and 1000 µg/mL), *C. albicans* ATCC 10231, and *C. albicans* CCC 191-13 (MICs of 1000 µg/mL). Only CSEAR12 showed activity against *C. parapsilosis* and *C. albicans* CCC 132-15 (MICs = 1000 µg/mL), while CSE10BR1 was only active against *C. glabrata* (MIC = 1000 µg/mL). For the antibiotics, the MICs of Ampicillin ranged from 0.25 to 32 µg/mL against the bacteria tested, while for Amphotericin B, the MICs ranged from 0.125 to 1 µg/mL against the different *Candida* strains.

### 3.3. Biofilm Inhibition

**Figure 3:**
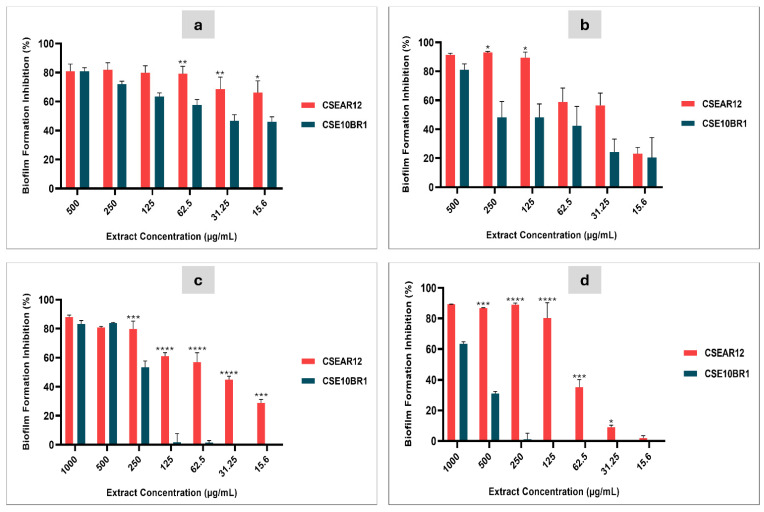
Biofilm formation inhibition by culture supernatant extracts from *T. afroharzianum* 10BR1 (CSE10BR1) and UEPA AR12 (CSEAR12) against *E. coli* (**a**), *E. faecalis* (**b**), *C. albicans* (**c**), and *C. parapsilosis* (**d**). Biofilm inhibition was measured using crystal violet staining for bacterial strains and the MTT assay for *Candida* strains in 96-well microplates. Data are presented as mean percentage inhibition ± standard error of the mean (SEM) from three independent experiments. Statistical significance between treatments was determined by two-way ANOVA followed by Tukey’s post-hoc test, with significance levels indicated by asterisks on the bars: *p* < 0.05 (*), *p* < 0.01 (**), *p* < 0.001 (***), and *p* < 0.0001 (****). Positive controls were Gentamicin (4 µg/mL), which inhibited biofilm formation by 52.6% ± 1.8% in *E. faecalis* and 70.3% ± 4.8% in *E. coli*, and Amphotericin B (2 µg/mL), which inhibited 91.94% ± 0.84% in *C. albicans* and 90.7% ± 0.66% in *C. parapsilosis*.

The effect of different concentrations of *T. afroharzianum* extracts was evaluated in 96-well polystyrene microplates, with biofilm formation detected using crystal violet for bacterial strains and MTT for *Candida* strains. Both extracts demonstrated activity against biofilm formation in the tested microorganisms, with CSEAR12 exhibiting higher activity than CSE10BR1 against all evaluated strains. Statistically significant differences between the extracts were observed from 62.5 to 15.6 µg/mL in *E. coli*; at 250 and 125 µg/mL in *E. faecalis*; from 250 to 15.6 µg/mL in *C. albicans*; and from 500 to 31.25 µg/mL in *C. parapsilosis* (two-way ANOVA, Tukey’s post hoc test, *p* < 0.05). The inhibitory effect decreased progressively with lower concentrations, reaching the minimum inhibitory activity at 15.6 μg/mL in most strains (Figure 3).

For CSEAR12, biofilm formation in *E. coli* was inhibited by more than 50% at all tested concentrations, suggesting that the IC_50_ is lower than the minimum tested concentration of 15.6 µg/mL. In contrast, for *E. faecalis*, CSEAR12 showed an IC_50_ of 28.23 µg/mL, with the highest inhibition observed at 250 µg/mL (92.8%), while inhibition in *E. coli* at the same concentration was 81.8%. For CSE10BR1, the IC_50_ against *E. coli* was 40.65 µg/mL, while against *E. faecalis*, it was 262.4 µg/mL. The highest inhibitions for CSE10BR1 were observed at the highest concentration tested, 500 µg/mL, with 81.5% inhibition for *E. coli* and 81.05% for *E. faecalis*.

In *C. albicans*, CSEAR12 exhibited an IC_50_ of 48.50 µg/mL, while for *C. parapsilosis*, the IC_50_ was 71.12 µg/mL. In contrast, CSE10BR1 had an IC_50_ of 241.5 µg/mL in *C. albicans* and 792.6 µg/mL in *C. parapsilosis*. Notably, the highest inhibition for all conditions was observed at 1000 µg/mL: CSEAR12 reached 87.9% inhibition in *C. albicans* and 89.3% in *C. parapsilosis*, while CSE10BR1 achieved 83.3% and 63.4% inhibition, respectively. All results were compared with the control without extract. The IC_50_ values for each strain are presented in Table 2. It is important to note that the highest concentration tested against the bacteria was 500 µg/mL, while for the *Candida* strains, it was 1000 µg/mL.

### 3.4. Antioxidant Activity

**Figure 4:**
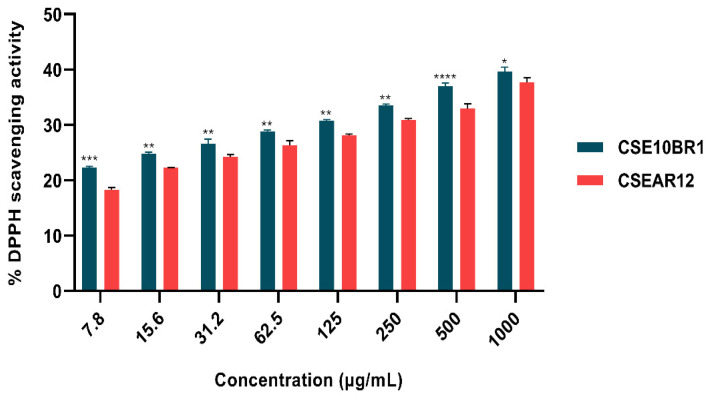
DPPH radical scavenging activity of *T. afroharzianum* culture supernatant extracts (CSE) at concentrations ranging from 7.8 to 1000 µg/mL. Data are presented as mean ± standard deviation from three independent experiments (n = 3). Statistical significance between CSE10BR1 and CSEAR12 at each concentration was determined by two-way ANOVA followed by Tukey’s post-hoc test, with significance indicated by asterisks: *p* < 0.05 (*), *p* < 0.01 (**), *p* < 0.001 (***), and *p* < 0.0001 (****). Maximum activity remained below 50% inhibition, suggesting that IC_50_ values are greater than 1000 µg/mL.

The antioxidant activity of *T. afroharzianum* extracts was evaluated at concentrations ranging from 7.8 to 1000 μg/mL using the DPPH assay, showing a progressive and concentration-dependent increase in radical scavenging activity (Figure 4). The scavenging percentages ranged from 12.88 ± 0.45% to 39.67 ± 0.78%, with CSE10BR1 consistently exhibiting significantly higher antioxidant activity compared to CSEAR12 at all tested concentrations (two-way ANOVA followed by Tukey’s post hoc test, *p* < 0.05). However, the maximum tested concentration (1000 μg/mL) was insufficient to reach 50% radical inhibition, indicating that the IC_50_ is greater than 1000 μg/mL. These results indicate that while both extracts possess antioxidant capacity, CSE10BR1 is more effective, although relatively high concentrations are required for substantial radical scavenging.

### 3.5. Cytotoxic Activity

**Figure 5:**
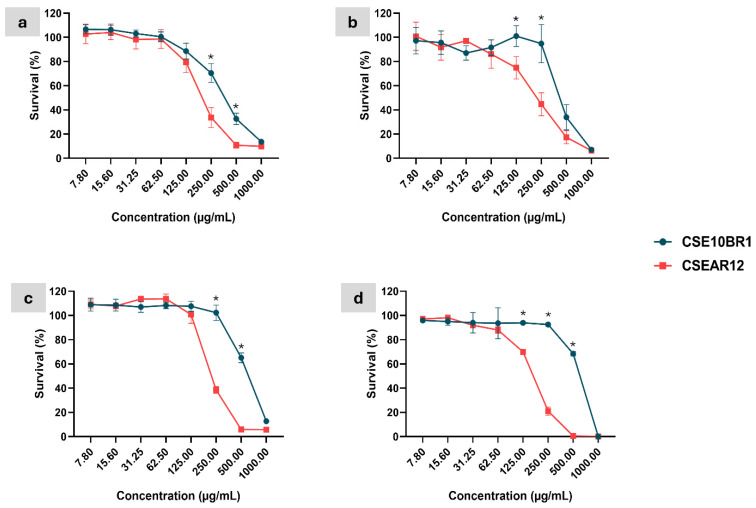
Cytotoxic effects of the culture supernatant extracts from *T. afroharzianum* strains 10BR1 (CSE10BR1) and UEPA AR12 (CSEAR12) on cell viability at increasing concentrations (7.8–1000 µg/mL) in four cell lines: (**a**) HaCaT, (**b**) HEK293, and (**c**) Vero—all non-tumor cell lines—and (**d**) Huh7, a tumor cell line. Statistical analysis was performed using two-way ANOVA followed by Tukey’s post-hoc test. Asterisks indicate statistically significant differences between extracts at each concentration (*p* < 0.05).

The determination of the IC_50_ for CSE10BR1 in HaCaT, HEK293, and Vero cells was 368.7 µg/mL, 437.8 µg/mL, and 593.7 µg/mL, respectively, while in Huh7 tumor cells, the IC_50_ was 602.1 µg/mL. In comparison, the CSEAR12 extract showed lower IC_50_ values across all tested cell lines: 202.5 µg/mL in HaCaT, 217.5 µg/mL in HEK293, 234.3 µg/mL in Vero, and 182.5 µg/mL in Huh7 (Figure 5), indicating higher cytotoxicity relative to CSE10BR1. Overall, Vero and Huh7 cells exhibited the highest IC_50_ values against CSE10BR1, suggesting lower sensitivity to this extract. For CSEAR12, the highest IC_50_ was also observed in Vero cells, while the lowest was recorded in Huh7. These results suggest that the Vero cell line has relatively greater resistance to the cytotoxic effects induced by the extracts, particularly to CSE10BR1.

When comparing both extracts within each cell type, statistically significant differences were observed based on two-way ANOVA followed by Tukey’s post-hoc test (*p* < 0.05). In HaCaT cells, CSE10BR1 showed significantly lower cytotoxicity than CSEAR12 at 250 and 500 µg/mL, while in HEK293 cells, differences were found at 125 and 250 µg/mL. In Vero cells, significant differences were detected at 250 and 500 µg/mL, and in Huh7 cells at 125, 250, and 500 µg/mL. These findings reinforce the lower cytotoxicity profile of the CSE10BR1 extract across all tested cell lines.

To assess the safety profile of both extracts, the SI was calculated as the ratio between IC_50_ and the MIC (SI = IC_50_/MIC). CSE10BR1 generally showed higher SI values than CSEAR12 in Huh7 and Vero cells, while in HEK293 and HaCaT cells, the values were comparable or variable depending on the pathogen. Against *S. aureus*, SI values > 10 across all cell lines for both extracts, indicating good selectivity and therapeutic potential [41], where SI > 10 is considered indicative of selective antimicrobial activity with low cytotoxicity. The highest SI for CSE10BR1 was 19.3 in Huh7 cells, followed by 19.0 in Vero cells, while for CSEAR12, the highest value was 15.0 in Vero cells. These results highlight the potential of both extracts to selectively target *S. aureus* with low cytotoxicity in mammalian cells. Against *E. faecalis*, SI values for CSE10BR1 ranged from 1.5 to 2.4, and for CSEAR12 from 1.5 to 1.9, indicating moderate selectivity. For *S. Typhimurium*, values were similar: 1.5 to 2.4 for CSE10BR1 and 0.7 to 0.9 for CSEAR12. In the case of *E. coli*, CSEAR12 showed consistently low SI values (0.36–0.47), while CSE10BR1 reached up to 1.20 in Huh7 cells, suggesting mild selectivity in some lines. Finally, against *Candida* spp., most SI values were below 1 for both extracts, indicating limited selectivity. Complete SI values are available in Supplementary Table S2.

### 3.6. Metabolite Identification by HPLC/MS

To gain insight into the metabolite composition responsible for these activities, we performed an HPLC-MS analysis. This enabled the identification of 21 metabolites previously reported in the literature. Of these, 20 compounds were identified in CSE10BR1 and 20 in CSEAR12 (Table 3). Atrichodermona C was detected exclusively in the CSEAR12 extract, while β-sitosterol appeared only in CSE10BR1, indicating strain-specific metabolic outputs.

Key compounds identified in both extracts included 6-pentyl-2-pyrone (6PP), 6-(pent-1-enyl)-pyran-2-one, 1,8-di-OH-3-methyl-anthraquinone, 6-methyl-1,3,8-trihydroxyanthraquinone, and koninginins, which are known for their bioactive properties. In addition, pachybasin (1-hydroxy-3-methyl-anthraquinone) and harzianopyridone were abundant in both strains, highlighting their potential role in antimicrobial or antioxidant applications. Other compounds such as β-sitosterol, ergosta-7,22-dien-3-ol, and 3,5,9-trihydroxyergosta-7,22-dien-6-one were detected at varying intensities in both strains, indicating a diverse metabolic profile that may contribute to synergistic bioactive effects.

Further details on the metabolites identified, including their classification into different metabolic groups, can be found in Supplementary Table S3.

### 3.7. Secondary Metabolite Biosynthesis in Trichoderma 10BR1 and UEPA AR12

**Figure 6:**
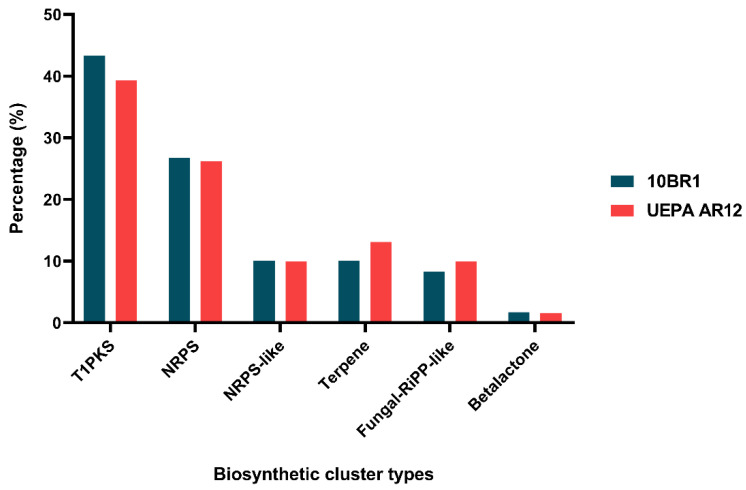
Distribution of secondary metabolite biosynthetic clusters in the genomes of *T. afroharzianum* strains 10BR1 and UEPA AR12 based on FUNGISMASH analysis. Paired bars represent the percentage of each biosynthetic cluster type found in each strain, allowing easy comparison. Percentages were calculated from the total occurrences of each cluster type across the identified genomic regions. Since some regions contain multiple biosynthetic pathways, each cluster type was counted separately.

The genome analysis of strain 10BR1 using the FUNGISMASH portal revealed the presence of 51 regions involved in the biosynthesis of secondary metabolites. Of these, 43.3% encode type I polyketide synthases (T1PKS), 26.7% encode non-ribosomal peptide synthetases (NRPS), 10% correspond to NRPS-like enzymes (related to NRPS but lacking complete elongation modules), 10% are associated with terpene biosynthesis, 8.3% encode fungal-RiPP-like compounds (ribosomally synthesized and enzymatically modified peptides), and 1.7% are linked to the biosynthesis of beta-lactones. Similarly, the genome of strain UEPA AR12 exhibited a high diversity of genes associated with secondary metabolite biosynthesis, with a total of 49 predicted regions. Among these, 39.3% encode T1PKS, 26.2% encode NRPS, 13.1% are linked to terpene biosynthesis, 9.9% correspond to NRPS-like enzymes, 9.9% encode fungal-RiPP-like compounds, and 1.6% are associated with beta-lactone biosynthesis (Figure 6).

Regarding metabolites with the highest similarity to known clusters (above 50%), the genome of strain 10BR1 revealed biosynthetic pathways for choline (100%), harzianopyridone (60%), the terpene clavarinic acid (100%), the polyketide harzifilone (90%), tricholignane A (77%), dichlorodiaportin (50%), and trichoxide (75%), along with the NRPS peramine (100%) and the terpene trichobrasilenol (60%). Meanwhile, the genome of strain UEPA AR12 contained pathways for the production of Metachelin C (including variants such as Metachelin A, A-CE, B, and dimerumic acids) (100%), harzianopyridone (60%), choline (100%), trichobrasilenol (including variants such as Brasilane A, F, E, and D) (60%), harzifilone (80%), and tricholignane A (77%) (Supplementary Tables S4 and S5).

## 4. Discussion

Strains 10BR1 and UEPA AR12 showed >99% identity in the *rpb2* gene with *T. afroharzianum*, confirming their taxonomic assignment [64]. The phylogenetic tree constructed from the gene sequences revealed that both strains clustered with other *T. afroharzianum* strains, forming a consistent group within the species. However, strain UEPA AR12 showed a closer relationship to specific isolates, such as GJS 04-186, GJS 00-24, and Tafum1, while strain 10BR1 was positioned more distantly, forming a unique subclade within the overall cluster. This distinct positioning likely reflects phylogenetic divergence, suggesting that strain 10BR1 represents a genetic variant within *T. afroharzianum*, although no functional differences have been assessed.

The first formal description of *T. afroharzianum* was associated with isolates from Africa, specifically from agricultural soils and related habitats, reflecting its native geographic origin. Since then, the species has been found in different regions of the world, demonstrating a broad ecological distribution. *T. afroharzianum* belongs to the *T. harzianum* species complex, which also includes *T. lixii*, *T. guizhouense*, and *T. harzianum* [65]. Studies on this complex have contributed to advancing the taxonomic resolution of the genus *Trichoderma*.

Many *Trichoderma* species, including those of endophytic origin, can produce a wide range of metabolites with diverse biological properties [66,67,68]. These bioactive compounds, particularly those from *T. afroharzianum* strains isolated in Argentina, emphasize the potential of *Trichoderma* species in clinical applications and contribute to a deeper understanding of *Trichoderma* biodiversity.

The antimicrobial activity of crude *Trichoderma* extracts has been widely documented, predominantly using agar diffusion methods. However, fewer studies report MIC values against key clinical pathogens. In our study, crude extracts from *T. afroharzianum* strains UEPA AR12 and 10BR1 exhibited notable inhibitory activity against *S. aureus* (MICs of 15.6–31.25 µg/mL), consistent with the 15.6 µg/mL reported by Nongthombam et al. [69] for *Trichoderma* sp. L2D2 isolated from *Anaphalis contorta*, and the 7.8–15.6 µg/mL range reported by Leylaie and Zafari [70] for endophytic *Trichoderma* spp. from *Vinca* species. Against *E. coli*, our MICs (500 µg/mL) were considerably higher than those reported in the same studies (7.8–15,6 µg/mL). These differences likely reflect variability in metabolite composition influenced by strain differences and cultivation or extraction conditions. For *E. faecalis*, our MICs (125–250 µg/mL) were comparable to the 125 µg/mL reported by Nongthombam et al. [69]. The MIC for *S. Typhimurium* in our study (250 µg/mL) was higher than the 31.25 µg/mL reported for *Salmonella enterica* serovar Typhi in the same study, indicating a comparatively moderate inhibitory effect against this serovar. The anti-yeast activity observed here (MIC = 250–1000 µg/mL against several *Candida* spp.) adds relevant data due to the limited MIC reports on crude *Trichoderma* extracts against yeasts. Although some studies describe MICs for purified fractions or isolated metabolites, such results are not directly comparable to those obtained with crude extracts.

The antimicrobial activity observed in the culture supernatant extracts of this study can be attributed to the presence of several metabolites identified in the HPLC/MS analysis of *T. afroharzianum* extracts. These include compounds such as anthraquinones (e.g., pachybasin), pyrones (e.g., 6PP), sterols (e.g., stigmasterol, β-sitosterol), pyrazine derivatives (e.g., harzianic acid), polyketides (e.g., koninginins), and other bioactive molecules such as asperelines and harzianopyridone, many of which have been previously reported to have antimicrobial properties.

Harzianic acid has demonstrated antimicrobial activity against both agricultural and clinical pathogens. Its efficacy has been confirmed against *F. oxysporum* (MIC: 0.125–0.250 mg/mL) [50] and *Staphylococcus pseudintermedius*, including both methicillin-resistant (MRSP, MIC: 32 µg/mL) and methicillin-sensitive (MSSP, MIC: 16 µg/mL) strains [51]. Recent studies indicate that its mode of action involves targeting the cell membrane of Gram-positive bacteria, causing structural disruption and membrane permeabilization [71]. Additionally, harzianic acid may interfere with essential metabolic processes, such as iron acquisition and branched-chain amino acid biosynthesis, by acting as an iron chelator and inhibitor of acetohydroxyacid synthase (AHAS) [72,73].

Stigmasterol is a steroid from the tetracyclic triterpenoid class structurally similar to cholesterol. This compound has been shown to have a wide range of biological properties, including anti-cancer, anti-arthritic, anti-inflammatory, anti-diabetic, immunomodulatory, anti-parasitic, antifungal, antibacterial, antioxidant, and neuroprotective activities [54]. In terms of antimicrobial activity, stigmasterol (at 100 µg/mL) was reported to exhibit inhibition zones of 29 mm against *S. aureus*, 24 mm against *E. coli*, and 25 mm against *C. albicans* [74]. Additionally, at a concentration of 50 µg/mL, it showed inhibition zones of 21 mm against *S. aureus*, 24 mm against *Bacillus subtilis*, 21 mm against *E. coli*, and 21 mm against *C. albicans* [75]. Mechanistically, stigmasterol integrates into microbial cell membranes, altering their fluidity and permeability. Such integration can disrupt membrane integrity, leading to increased ion leakage and impaired cellular function [76].

Another interesting compound identified was 6PP, an aromatic substance known for its characteristic coconut-like scent and prominent antifungal properties [18]. Its activity has been well documented against several phytopathogens, including *F. oxysporum*, *Rhizoctonia solani* [77], and *B. cinerea* [78]. This volatile compound induces oxidative stress in fungal pathogens by promoting the generation of reactive oxygen species (ROS), such as superoxide radicals and hydrogen peroxide. The accumulation of ROS overwhelms the pathogen’s antioxidant defenses, leading to cellular damage and death [79]. In addition, the bioactive analogue 6-(pent-1-enyl)pyran-2-one, also identified in this study, has shown antifungal activity against clinically relevant fungi, such as *C. albicans*, *Penicillium* spp., *Cryptococcus neoformans*, and *Aspergillus fumigatus* [42]. Furthermore, another study reported its antifungal activity against *R. solani*, *Sclerotium rolfsii*, *M. phaseolina*, and *F. oxysporum*, with EC_50_ (the effective concentration required for 50% inhibition of mycelial growth) values of 64.6 µg/mL, 38.8 µg/mL, 90.6 µg/mL, and 74.3 µg/mL, respectively [43].

β-sitosterol, a widely recognized phytosterol, exhibits a wide range of medicinal properties, including anti-inflammatory, antihypertensive, and antibacterial activities [59]. Previous studies have shown that β-sitosterol has antibacterial activity against various bacterial species, such as *S. aureus* and *E. coli* [59,60]. Furthermore, another study reported significant antifungal activity against *R. solanacearum*, with an MIC value of 31.3 μg/mL [61].

Pachybasin (1-hydroxy-3-methyl-anthraquinone) has been identified as a compound with potent antimicrobial activity, effective against a wide range of microorganisms, including *E. coli*, *S. aureus*, *B. subtilis*, *Micrococcus luteus*, *C. albicans*, *Saccharomyces cerevisiae*, *Aspergillus niger*, *Aspergillus flavus*, and *F. oxysporum* [47]. Another identified compound, harzianopyridone, has shown significant antifungal activity against *R. solani*, *Sclerotium rolfsii*, *M. phaseolina*, and *F. oxysporum*, with EC_50_ values of 35.9 µg/mL, 42.2 µg/mL, 60.4 µg/mL, and 50.2 µg/mL, respectively [43]. In addition, the compound 3,5,9-trihydroxyergosta-7,22-dien-6-one, present in CSEAR12, was isolated from *Trichoderma* spp. by Xuan et al. [62] and showed antimicrobial properties against *E. coli*, *B. subtilis*, *Pyricularia oryzae*, *C. albicans*, *A. niger*, and *Alternaria alternata*, with MICs of 512 µg/mL for *E. coli*, 256 µg/mL for *B. subtilis*, and 32 µg/mL for the remaining pathogens. These results highlight the diversity of bioactive compounds present in *T. afroharzianum* extracts and reinforce their potential for the development of novel antimicrobial agents.

In addition to its antimicrobial effects, this study demonstrated that crude extracts of *T. afroharzianum* exhibit strong antibiofilm activity. Quantitative data on *Trichoderma* extracts with similar antibiofilm effects remain limited, particularly against the strains tested in our study. However, some comparisons can be drawn. For instance, an ethyl acetate extract from the endophytic strain *T. virens* BLR24 inhibited MRSA biofilms by 51.9% at 50 µg/mL [80], a level comparable to that achieved by CSEAR12 against *E. faecalis* (56.6% at 31.25 µg/mL) and *E. coli* (66.3% at 15.6 µg/mL). Regarding extracts from other fungi, our results also present relevant parallels. For example, the crude extract of *Aspergillus* sp. AP5, isolated from *P. australis* leaves, showed less than 50% inhibition of *E. coli* biofilm formation at 500 µg/mL [81], while our CSEAR12 extract achieved 66.3% inhibition at only 15.6 µg/mL, indicating greater efficacy. Additionally, the crude extract of the endophytic fungus *Chaetomium globosum*, isolated from *Moringa oleifera*, inhibited *C. albicans* biofilm formation by approximately 50% [82], a result comparable to that of CSEAR12, which reached 56.8% inhibition at 62.5 µg/mL.

In direct comparison with standard antibiotics, CSEAR12 inhibited *E. coli* biofilms by 66.26% at 15.6 µg/mL, closely approaching the inhibition achieved by Gentamicin at 4 µg/mL (70.3%). Against *E. faecalis*, CSEAR12 reached 56.6% inhibition at 31.25 µg/mL, while CSE10BR1 inhibited only 24.6% at the same concentration. In terms of IC_50_ values, CSEAR12 showed an IC_50_ of 28.2 µg/mL for *E. faecalis*, which is consistent with reported Gentamicin activity in the literature (e.g., ~45–50% inhibition at 8 µg/mL [83]. Regarding yeasts, CSEAR12 inhibited *C. albicans* and *C. parapsilosis* biofilms by 56.8% and 35.1%, respectively, at 62.5 µg/mL. At the highest tested concentration (1000 µg/mL), inhibition reached 87.9% in *C. albicans* and 89.3% in *C. parapsilosis*, with IC_50_ values of 48.5 µg/mL and 71.1 µg/mL, respectively (Figure 3). These effects closely approach those of Amphotericin B at 2 µg/mL, which inhibited *C. albicans* and *C. parapsilosis* biofilms by 91.94% ± 0.84% and 90.7% ± 0.66%, respectively.

These comparisons highlight that although crude extracts require higher concentrations than purified drugs or isolated fungal metabolites, they can achieve similar levels of biofilm inhibition. For example, the isolated compound 6PP produced by *T. atroviride* P1, reduced *Xanthomonas campestris* biofilms by ~70% at just 0.001 µg/mL when combined with a specific phage [44], while harzianic acid showed dose-dependent inhibition of MRSP and MSSP biofilms starting at 16 µg/mL [51]. Altogether, our findings reinforce the potential of *Trichoderma* spp. as sources of both individual antibiofilm compounds and complex metabolite mixtures, which may act synergistically to disrupt microbial communities.

The antioxidant activity of the culture supernatants was investigated in this study, showing a statistically significantly higher activity for CSE10BR1 compared to CSEAR12 at all tested concentrations, with a maximum activity of 39.67 ± 0.78% at 1000 µg/mL. This result is consistent with the findings of Saravanakumar et al. [84], who reported a DPPH radical scavenging activity of 37.25 ± 2.25% at 500 µg/mL for a *T. atroviride* extract. However, Narendran and Kathiresan [85] recorded even higher antioxidant activity, with a maximum DPPH radical scavenging of 54.61% at 160 µg/mL for an ethyl acetate extract from a mangrove-origin *Trichoderma* sp. strain.

Although the maximum tested concentration (1000 µg/mL) did not reach 50% inhibition, indicating that the IC_50_ is greater than 1000 µg/mL, the significant radical scavenging activity observed highlights the ability of molecules present in the fungal extracts to neutralize these radicals [86]. This antioxidant activity may be related to the presence of several secondary metabolites identified by HPLC/MS, particularly compounds with previously reported antioxidant activity, such as 6PP [45], stigmasterol [87,88], and β-sitosterol [89], as well as some anthraquinones, including Pachybasin and 6-methyl-1,3,8-trihydroxyanthraquinone [39]. These findings underline the potential of these extracts for applications in medicine and nutritional therapy.

In this study, we found that the CSE10BR1 extract exhibited lower cytotoxicity compared to CSEAR12, as confirmed by statistical analysis, supporting its favorable safety profile across different cell lines. The SI calculated for both extracts showed values > 10 against *S. aureus* in all tested cell lines, indicating good selectivity and therapeutic potential. CSE10BR1 displayed the highest SI values, although CSEAR12 also achieved SI values above 10 against *S. aureus*, demonstrating that both extracts can selectively target this bacterium with low toxicity to mammalian cells. For other pathogens such as *E. faecalis* and *S. Typhimurium*, the SI values indicated moderate to low selectivity, while for *E. coli* and *Candida* spp., selectivity was limited. This was mainly due to the low antimicrobial potency of the extracts against these organisms, as shown by high MIC values (mostly 500–1000 µg/mL), along with some cytotoxicity at these concentrations. Thus, although both extracts show potential against Gram-positive bacteria, particularly *S. aureus*, their use against certain Gram-negative bacteria and yeasts appears less promising.

Despite the observed differences in cytotoxicity and selectivity, both extracts may have distinct applications depending on the concentration used and the context of use. CSE10BR1, with its lower toxicity and higher selectivity, stands out as a promising candidate for the development of antibacterial products, particularly in topical or non-systemic formulations. On the other hand, although CSEAR12 exhibited higher cytotoxicity, it may still be explored at lower concentrations or in specific contexts, provided that further studies confirm its safety. It is essential to advance the identification and characterization of the bioactive compounds present in these extracts to better understand their mechanisms of action and improve their selectivity. While this study included testing on a human tumor cell line (Huh7), additional evaluations using other cell lines and animal models will be necessary to confirm the therapeutic potential of these extracts and to assess their safety and efficacy in clinical or commercial settings.

The genome analysis of *T. afroharzianum* strains 10BR1 and UEPA AR12 revealed a diverse array of gene clusters associated with secondary metabolite biosynthesis, including polyketides, non-ribosomal peptides, and terpenes, underscoring their potential for producing bioactive compounds. These findings are consistent with previous studies indicating that T1PKS and NRPS are the most frequently predicted core enzymes in *Trichoderma* species [90]. The predominance of T1PKS suggests a robust capacity for polyketide synthesis, a class of compounds widely recognized for their antimicrobial properties [91,92]. Furthermore, the presence of NRPS and NRPS-like enzymes highlights the strains’ ability to synthesize non-ribosomal peptides, which are known for their structural and functional diversity, including roles as antibiotics and siderophores [93].

Genomic regions encoding enzymes involved in terpene biosynthesis were also identified, suggesting a role for these compounds in plant–microbe communication, PGP, and pathogen resistance [94,95]. In addition, terpenes may exhibit cytotoxic and antioxidant activities, expanding the potential biotechnological applications of these strains [96]. The presence of genes associated with betalactone synthesis and fungal-RiPP-like compounds further underscores the strains’ capacity to produce metabolites with antibiotic, antiparasitic, and cytotoxic properties [97,98].

The integration of FUNGISMASH and HPLC/MS data enabled the correlation of predicted biosynthetic pathways with experimentally identified compounds. Harzianic acid is likely associated with the hybrid T1PKS-NRPS clusters found in the genome, although it was not directly predicted by FUNGISMASH. Similarly, stigmasterol was also not predicted. However, its biosynthesis may be linked to terpene pathways detected in the genome, since sterols share common precursors with this compound class.

In addition to the biosynthetic pathways discussed earlier, FUNGISMASH analysis identified gene clusters with high similarity (>50%) to known pathways for producing metabolites such as harzianopyridone, clavaric acid, harzifilone, and trichobrasilenol, among others. Harzianopyridone, whose presence was confirmed by HPLC/MS, corresponds to the prediction of a hybrid NRPS–PKS cluster by FUNGISMASH. Although the other compounds were not experimentally detected in this study, their predicted presence indicates further potential for the synthesis of bioactive metabolites. Importantly, the genomic analysis also included a targeted search for genes involved in mycotoxin biosynthesis. We specifically screened for homologs of the trichodiene synthase gene (*TRI5*), the first and essential enzyme in the trichothecene biosynthetic pathway, using sequences from *T. harzianum* (Q6A1B7), *Trichoderma brevicompactum* (F4MJM0), and *Trichoderma arundinaceum* (G0LES5) as references. No homologous coding sequences for *TRI5* were identified in the genomes of strains 10BR1 and UEPA AR12. Although homologs of trichothecene 3-O-acetyltransferase genes were detected, these are likely involved in biosynthetic pathways unrelated to trichothecene production. Correspondingly, HPLC-MS analysis did not detect any known mycotoxins or related metabolites, supporting a low risk of mycotoxin production by these strains under the tested conditions.

To further advance the translational potential of our findings, future work should prioritize the isolation and structural characterization of the key bioactive metabolites responsible for the observed biological properties. Once purified, these compounds should be evaluated individually and in defined combinations to elucidate their mechanisms of action and possible synergistic interactions. In parallel, in vivo efficacy and safety should be assessed using relevant animal models of infection, including biofilm-associated infections, to provide critical insight into pharmacokinetics, toxicity, and therapeutic dosage. Finally, formulation strategies such as encapsulation in nanoparticles, incorporation into hydrogels, or development of surface coatings should be explored to enhance compound stability, target delivery to infection sites, and prevent biofilm establishment on medical devices. Our group’s previous experience with nanoparticle formulations of bioactive plant extracts supports the feasibility of applying similar strategies to these metabolites [99]. Collectively, these approaches will not only validate the clinical utility of *T. afroharzianum* metabolites but also lay the groundwork for novel antifouling and anti-infective products.

## 5. Conclusions

The two *T. afroharzianum* strains evaluated here proved to be rich sources of bioactive metabolites, displaying antimicrobial, antibiofilm, and antioxidant activities. Both extracts were most effective against *S. aureus*, with SI >10 across all cell lines, supporting their potential for targeted antibacterial applications, although moderate to limited selectivity against other pathogens, especially *E. coli* and *Candida* spp., indicates some cytotoxicity at higher concentrations. In biofilm assays, CSEAR12 stood out with stronger inhibition of *E. coli*, *E. faecalis*, *C. albicans*, and *C. parapsilosis*. HPLC/MS profiling revealed a diverse array of anthraquinones, pyranones, sterols, pyrazines, and other bioactive compounds. Genomic analysis via FUNGISMASH uncovered a broad repertoire of secondary metabolite biosynthetic gene clusters, predominantly polyketide synthases, non-ribosomal peptide synthetases, and terpene synthases, highlighting the genetic potential of these strains to serve as microbial biofactories for producing structurally diverse antimicrobial compounds. Importantly, targeted genomic searches confirmed the absence of mycotoxin-related genes, such as *TRI5*, further supporting the safety profile of these fungi as metabolite producers.

Taken together, these results underscore the promise of *Trichoderma* species not only as sources of novel antimicrobial agents but also as versatile platforms for sustainable biotechnological production of bioactive metabolites. Future research should focus on the isolation and structural characterization of key metabolites, in vivo efficacy and safety assessments, and the development of optimized formulations to translate these findings into practical pharmaceutical and industrial applications.

## Figures and Tables

**Table 1:** Minimum inhibitory concentration (MIC) of culture supernatant extracts from two strains of *T. afroharzianum* against various microorganisms using the broth microdilution assay.

Strains	Extracts (µg/mL)		Amp	Amb
	CSE10BR1	CSEAR12		
Bacteria:				
*S. aureus* (ATCC 25923)	**31.25**	**15.6**	0.25	-
*E. faecalis* (ATCC 29212)	250	125	0.25	-
*E. coli* (ATCC 25922)	500	500	2	-
*E. coli* (ATCC 35218)	500	500	32	-
*Salmonella Typhimurium* (ATCC 14028s)	250	250	0.5	-
Fungi:				
*C. albicans* (ATCC 10231)	1000	1000	-	0.125
*C. parapsilosis* (ATCC 22019)	>1000	1000	-	0.125
*C. tropicalis* (ATCC 200956)	1000	500	-	1
*C. krusei* (ATCC 6258)	500	**250**	-	0.5
*C. glabrata* (ATCC 2950)	1000	>1000	-	1
*C. albicans* (CCC 191-13)	1000	1000	-	0.5
*C. albicans* (CCC 132-15)	>1000	1000	-	0.25

CSE: Culture Supernatant Extracts; Amp: Ampicillin; Amb: Amphotericin B.

**Table 2:** IC_50_ values for biofilm formation inhibition by *T. afroharzianum* extracts against different microorganisms.

Microorganism	Extracts (µg/mL)	
	CSE10BR1	CSEAR12
*E. coli*	40.65	<15.6
*E. faecalis*	262.4	28.23
*C. albicans*	241.5	48.50
*C. parapsilosis*	792.6	71.12

Note: The maximum tested concentration was 500 µg/mL for bacterial strains and 1000 µg/mL for *Candida* species, based on preliminary susceptibility assays indicating different tolerance levels between bacteria and yeasts.

**Table 3:** Compounds identified by HPLC/MS in the culture supernatant extracts of the two *T. afroharzianum* strains.

N°	*m/z*	Adduct	Metabolites ^1^	Molecular Formula	Biological Activity	References ^2^
1	165.0	M + H	6-(pent-1-enyl)pyran-2-one	C_10_ H_12_ O_2_	Antifungal	[42,43]
2	167.0	M + H	6-pentyl-2-pyrone	C_10_ H_14_ O_2_	Antifungal, antibiofilm, antioxidant, plant growth promoter	[18,44,45,46]
3	239.0	M + H	1-hydroxy-3-methyl-anthraquinone (Pachybasin)	C_15_ H_10_ O_3_	Antimicrobial, antioxidant	[47,48]
4	257.0	M + H	1,8-di-OH-3-methyl-anthraquinone	C_15_ H_12_ O_4_	Antifungal	[43]
5	271.1	M + H	6-methyl-1,3,8-trihydroxyanthraquinone	C_15_ H_10_ O_5_	Antifungal, antioxidant	[43,48]
6	282.0	M + H	Harzianopyridone	C_14_ H_19_ N O_5_	Antifungal	[43]
7	283.0	M + H	Koninginin E/B	C_16_ H_26_ O_4_	Antifungal	[18]
8	285.0	M + H	Koninginin A	C_16_ H_28_ O_4_	Antifungal	[18]
9	317.2	M + H	Trichodermaerin	C_20_ H_28_ O_3_	Antitumor	[49]
10	365.0	M + H	Harzianic acid	C_19_ H_27_ N O_6_	Antimicrobial, antibiofilm, plant growth promoter	[50,51,52]
11	353.0	M + H	Saturnispol C	C_22_ H_24_ O_4_	-	[53]
12	395.5	M + H	Stigmasterol	C_29_ H_48_ O	Antifungal, antibacterial, antioxidant, antitumor, anti-inflammatory, antidiabetic, immunomodulatory, antiparasitic, and neuroprotective activities	[54]
13	496.0	M + H	Trichotetronine	C_28_ H_32_ O_8_	-	[53]
14	295.2	M − H	Atrichodermone C	C_15_ H_24_ O_2_	Cytotoxic, anti-inflammatory	[55]
15	299.2	M − H	Methylcordysinin A	C_12_ H_20_ N_2_ O_3_	-	[56]
16	397.3	M − H	Ergosta-7,22-dien-3-ol	C_28_ H_46_ O	Antifungal, Antitumor	[57,58]
17	413.4	M − H	β-sitosterol	C_29_ H_50_ O	Antibacterial, antifungal, antioxidant, anti-inflammatory, antihypertensive	[18,59,60,61]
18	489.3	M − H	3,5,9-trihydroxyergosta-7,22-dien-6-one	C_28_ H_44_ O_4_	Antimicrobial	[62]
19	935.6	M − H	Aspereline A	C_45_ H_80_ N_10_ O_11_	Antibacterial	[63]
20	951.6	M − H	Aspereline E	C_45_ H_80_ N_10_ O_12_	Antibacterial	[63]
21	977.6	M − H	Aspereline H	C_47_ H_82_ N_10_ O_12_	Antibacterial	[63]

^1^ Atrichodermona C was identified exclusively in the CSEAR12 extract, whereas β-sitosterol was detected only in CSE10BR1. ^2^ The references indicate previously reported bioactivities for each compound. When no activity is listed, the references pertain solely to the prior identification of the compound in *Trichoderma* species.

## Data Availability

The original contributions presented in this study are included in the article/Supplementary Material. Further inquiries can be directed to the corresponding author.

## Supplementary Materials

The following supporting information can be downloaded at: https://www.mdpi.com/article/10.3390/jof11060457/s1. Table S1: Percentage of identity for the *rpb2* gene of strains 10BR1 and UEPA AR12 compared to other *Trichoderma* strains based on BLASTn analysis; Figure S1: Phylogenetic tree of *T. afroharzianum* strains, including all isolates from the study (10BR1 [GenBank: JBDIXD000000000], UEPA AR12 [GenBank: JBDIXE000000000], and others), with *Rhizoctonia endophytica* DAOM 138188 as the outgroup; Table S2: Selectivity index (SI = IC_50_/MIC) of *T. afroharzianum* extracts CSE10BR1 and CSEAR12 against microbial strains based on different human cell lines; Figure S2: HPLC-MS chromatograms of the main metabolites detected in the culture supernatant extract of *T. afroharzianum* strain 10BR1 (CSE10BR1); Figure S3: HPLC-MS chromatograms of the main metabolites detected in the culture supernatant extract of *T. afroharzianum* strain UEPA AR12 (CSEAR12); Table S3: Putative biosynthetic pathways for metabolites identified by HPLC-MS, based on FUNGISMASH genome analysis. Table S4: Genomic regions associated with secondary metabolite biosynthesis in *T. afroharzianum* 10BR1 according to FUNGISMASH; Table S5: Genomic regions associated with secondary metabolite biosynthesis in *T. afroharzianum* UEPA AR12 according to FUNGISMASH.

## Abbreviations

The following abbreviations are used in this manuscript:

AMP	Ampicillin
AMB	Amphotericin B
ATCC	American Type Culture Collection
CEFOBI	Centro de Estudios Fotosintéticos y Bioquímicos
CEREMIC	Mycological Reference Center
CFU	Colony-Forming Units
CSE	Culture Supernatant Extract
CSE10BR1	Culture Supernatant Extract from strain 10BR1
CSEAR12	Culture Supernatant Extract from strain AR12
DMSO	Dimethyl Sulfoxide
DMEM	Dulbecco’s Modified Eagle Medium
DPPH	2,2-Diphenyl-1-picrylhydrazyl
EC	Extract Control
FBS	Fetal Bovine Serum
GC	Growth Control
HPLC/MS	High-Performance Liquid Chromatography/Mass Spectrometry
MIC	Minimum Inhibitory Concentration
MOPS	3-(N-morpholino)propanesulfonic acid
MRSP	Methicillin-Resistant *Staphylococcus pseudintermedius*
MSSP	Methicillin-Sensitive *Staphylococcus pseudintermedius*
MTT	3-(4,5-Dimethylthiazol-2-yl)-2,5-diphenyltetrazolium bromide
NRPS	Non-Ribosomal Peptide Synthetase
OD	Optical Density
PBS	Phosphate-Buffered Saline
PDA	Potato Dextrose Agar
PDB	Potato Dextrose Broth
PGP	Plant-Growth Promotion
SC	Sterility Control
SD	Standard Deviation
SI	Selectivity Index
STR	Short Tandem Repeat
T1PKS	Type I Polyketide Synthase
TTC	Triphenyltetrazolium Chloride
YPD	Yeast Extract Peptone Dextrose medium
