# Supplementary material for: Exploring the Bioactive Secondary Metabolites of Two Argentine Trichoderma afroharzianum Strains
Source: J Fungi (Basel). 2025 Jun 17;11(6):457. doi: 10.3390/jof11060457 (PMC12194781; doi:10.3390/jof11060457)
Supplement: Supplementary file 1 [file jof-11-00457-s001.zip › Figure S1. Phylogenetic tree of T. afroharzianum strains, including all isolates from the study (10BR1, UEPA AR12, and others).pdf]

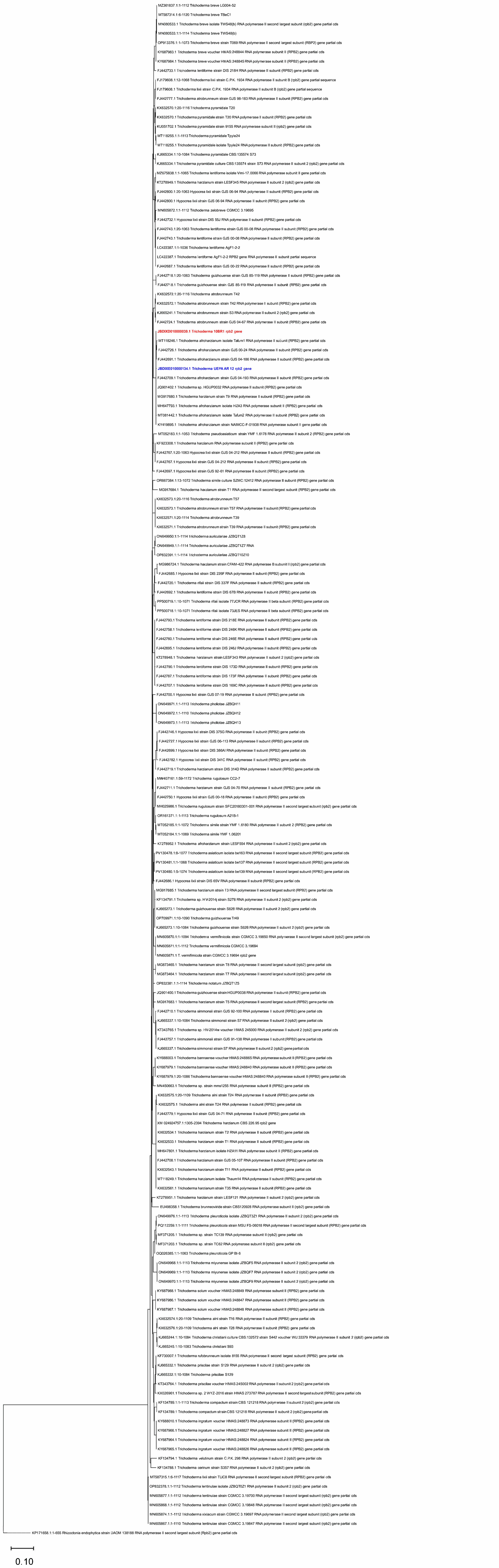
MZ361837.1:1-1112 Trichoderma breve LG004-52
MT587314.1:6-1120 Trichoderma breve TBeC1
MN080533.1 Trichoderma breve isolate TWS48(b) RNA polymerase II second largest subunit (rpb2) gene partial cds
MN080533.1:1-1114 Trichoderma breve TWS48(b)
OP913376.1:1-1073 Trichoderma breve strain T069 RNA polymerase II second largest subunit (RPB2) gene partial cds
KY687983.1 Trichoderma breve voucher HMAS:248844 RNA polymerase subunit II (RPB2) gene partial cds
KY687984.1 Trichoderma breve voucher HMAS:248845 RNA polymerase subunit II (RPB2) gene partial cds
FJ442733.1 Trichoderma lentiforme strain DIS 218H RNA polymerase II subunit (RPB2) gene partial cds
FJ179608.1:12-1068 Trichoderma lixii strain C.P.K. 1934 RNA polymerase II subunit B (rpb2) gene partial sequence
FJ179608.1 Trichoderma lixii strain C.P.K. 1934 RNA polymerase II subunit B (rpb2) gene partial sequence
FJ442777.1 Trichoderma atrobrunneum strain GJS 98-183 RNA polymerase II subunit (RPB2) gene partial cds
KX632570.1:20-1116 Trichoderma pyramidale T20
KX632570.1 Trichoderma pyramidale strain T20 RNA polymerase II subunit (RPB2) gene partial cds
KU051702.1 Trichoderma pyramidale strain 9155 RNA polymerase subunit II (rpb2) gene partial cds
MT118255.1:1-1113 Trichoderma pyramidale Tpyle24
MT118255.1 Trichoderma pyramidale isolate Tpyle24 RNA polymerase II subunit (RPB2) gene partial cds
KJ665334.1:10-1084 Trichoderma pyramidale CBS:135574 S73
KJ665334.1 Trichoderma pyramidale culture CBS:135574 strain S73 RNA polymerase II subunit 2 (rpb2) gene partial cds
MZ675838.1:1-1065 Trichoderma lentiforme isolate Vmi-17.0066 RNA polymerase subunit II gene partial cds
KT278949.1 Trichoderma harzianum strain LESF345 RNA polymerase II subunit 2 (rpb2) gene partial cds
FJ442800.1:20-1063 Hypocrea lixii strain GJS 06-94 RNA polymerase II subunit (RPB2) gene partial cds
FJ442800.1 Hypocrea lixii strain GJS 06-94 RNA polymerase II subunit (RPB2) gene partial cds
MN605872.1:1-1112 Trichoderma zelobreve CGMCC 3.19695
FJ442732.1 Hypocrea lixii strain DIS 55J RNA polymerase II subunit (RPB2) gene partial cds
FJ442743.1:20-1063 Trichoderma lentiforme strain GJS 00-08 RNA polymerase II subunit (RPB2) gene partial cds
FJ442743.1 Trichoderma lentiforme strain GJS 00-08 RNA polymerase II subunit (RPB2) gene partial cds
LC422387.1:1-1036 Trichoderma lentiforme AgF1-2-2
LC422387.1 Trichoderma lentiforme AgF1-2-2 RPB2 gene RNA polymerase II subunit partial sequence
FJ442687.1 Trichoderma lentiforme strain GJS 00-22 RNA polymerase II subunit (RPB2) gene partial cds
FJ442718.1:20-1063 Trichoderma guizhouense strain GJS 85-119 RNA polymerase II subunit (RPB2) gene partial cds
FJ442718.1 Trichoderma guizhouense strain GJS 85-119 RNA polymerase II subunit (RPB2) gene partial cds
KX632572.1:20-1116 Trichoderma atrobrunneum T42
KX632572.1 Trichoderma atrobrunneum strain T42 RNA polymerase II subunit (RPB2) gene partial cds
KJ665241.1 Trichoderma atrobrunneum strain S3 RNA polymerase II subunit 2 (rpb2) gene partial cds
FJ442724.1 Trichoderma atrobrunneum strain GJS 04-67 RNA polymerase II subunit (RPB2) gene partial cds
JBDIXD010000039.1 Trichoderma 10BR1 rpb2 gene
MT118246.1 Trichoderma afroharzianum isolate Tafum1 RNA polymerase II subunit (RPB2) gene partial cds
FJ442726.1 Trichoderma afroharzianum strain GJS 00-24 RNA polymerase II subunit (RPB2) gene partial cds
FJ442691.1 Trichoderma afroharzianum strain GJS 04-186 RNA polymerase II subunit (RPB2) gene partial cds
JBDIXE010000134.1 Trichoderma UEPA AR 12 rpb2 gene
FJ442709.1 Trichoderma afroharzianum strain GJS 04-193 RNA polymerase II subunit (RPB2) gene partial cds
JQ901402.1 Trichoderma sp. HGUP0032 RNA polymerase II subunit (RPB2) gene partial cds
MG917680.1 Trichoderma harzianum strain T9 RNA polymerase II subunit (RPB2) gene partial cds
MH647793.1 Trichoderma afroharzianum isolate HZA3 RNA polymerase subunit II (RPB2) gene partial cds
MT081442.1 Trichoderma afroharzianum isolate Tafum2 RNA polymerase II subunit (RPB2) gene partial cds
KY419895.1 Trichoderma afroharzianum strain NAIMCC-F-01938 RNA polymerase subunit II gene partial cds
MT052183.1:1-1053 Trichoderma pseudoasiaticum strain YMF 1.6178 RNA polymerase II subunit 2 (RPB2) gene partial cds
KF923308.1 Trichoderma harzianum RNA polymerase subunit II (RPB2) gene partial cds
FJ442767.1:20-1063 Hypocrea lixii strain GJS 04-212 RNA polymerase II subunit (RPB2) gene partial cds
FJ442767.1 Hypocrea lixii strain GJS 04-212 RNA polymerase II subunit (RPB2) gene partial cds
FJ442697.1 Hypocrea lixii strain GJS 92-61 RNA polymerase II subunit (RPB2) gene partial cds
OR667384.1:13-1072 Trichoderma simile culture SZMC:12412 RNA polymerase II subunit (RPB2) gene partial cds
MG917684.1 Trichoderma harzianum strain T1 RNA polymerase II second largest subunit (RPB2) gene partial cds
KX632573.1:20-1116 Trichoderma atrobrunneum T57
KX632573.1 Trichoderma atrobrunneum strain T57 RNA polymerase II subunit (RPB2) gene partial cds
KX632571.1:20-1114 Trichoderma atrobrunneum T39
KX632571.1 Trichoderma atrobrunneum strain T39 RNA polymerase II subunit (RPB2) gene partial cds
ON649950.1:1-1114 Trichoderma auriculariae JZBQT1Z8
ON649949.1:1-1114 Trichoderma auriculariae JZBQT1Z7 RNA
OP832391.1:1-1114 Trichoderma auriculariae JZBQT10Z10
MG986724.1 Trichoderma harzianum strain CFAM-422 RNA polymerase II subunit II (rpb2) gene partial cds
FJ442685.1 Hypocrea lixii strain DIS 226F RNA polymerase II subunit (RPB2) gene partial cds
FJ442720.1 Trichoderma rifaii strain DIS 337F RNA polymerase II subunit (RPB2) gene partial cds
FJ442692.1 Trichoderma lentiforme strain DIS 67B RNA polymerase II subunit (RPB2) gene partial cds
PP500719.1:10-1071 Trichoderma rifaii isolate 77JCR RNA polymerase II beta subunit (RPB2) gene partial cds
PP500718.1:10-1071 Trichoderma rifaii isolate 73JES RNA polymerase II beta subunit (RPB2) gene partial cds
FJ442793.1 Trichoderma lentiforme strain DIS 218E RNA polymerase II subunit (RPB2) gene partial cds
FJ442758.1 Trichoderma lentiforme strain DIS 246K RNA polymerase II subunit (RPB2) gene partial cds
FJ442760.1 Trichoderma lentiforme strain DIS 246E RNA polymerase II subunit (RPB2) gene partial cds
FJ442695.1 Trichoderma lentiforme strain DIS 246J RNA polymerase II subunit (RPB2) gene partial cds
KT278948.1 Trichoderma harzianum strain LESF343 RNA polymerase II subunit 2 (rpb2) gene partial cds
FJ442790.1 Trichoderma lentiforme strain DIS 173D RNA polymerase II subunit (RPB2) gene partial cds
FJ442787.1 Trichoderma lentiforme strain DIS 173F RNA polymerase II subunit (RPB2) gene partial cds
FJ442707.1 Trichoderma lentiforme strain DIS 169C RNA polymerase II subunit (RPB2) gene partial cds
FJ442700.1 Hypocrea lixii strain GJS 07-19 RNA polymerase II subunit (RPB2) gene partial cds
ON649971.1:1-1113 Trichoderma pholiotae JZBQH11
ON649972.1:1-1110 Trichoderma pholiotae JZBQH12
ON649973.1:1-1113 Trichoderma pholiotae JZBQH13
FJ442746.1 Hypocrea lixii strain DIS 375G RNA polymerase II subunit (RPB2) gene partial cds
FJ442727.1 Hypocrea lixii strain GJS 06-113 RNA polymerase II subunit (RPB2) gene partial cds
FJ442699.1 Hypocrea lixii strain DIS 386AI RNA polymerase II subunit (RPB2) gene partial cds
FJ442782.1 Hypocrea lixii strain DIS 341C RNA polymerase II subunit (RPB2) gene partial cds
FJ442719.1 Trichoderma harzianum strain DIS 314D RNA polymerase II subunit (RPB2) gene partial cds
MW407161.1:59-1172 Trichoderma rugulosum CC2-7
FJ442711.1 Trichoderma harzianum strain GJS 04-70 RNA polymerase II subunit (RPB2) gene partial cds
FJ442750.1 Hypocrea lixii strain GJS 00-18 RNA polymerase II subunit (RPB2) gene partial cds
MH025986.1 Trichoderma rugulosum strain SFC20180301-001 RNA polymerase II second largest subunit (rpb2) gene partial cds
OR161371.1:1-1113 Trichoderma rugulosum A21B-1
MT052185.1:1-1072 Trichoderma simile strain YMF 1.6180 RNA polymerase II subunit 2 (RPB2) gene partial cds
MT052184.1:1-1069 Trichoderma simile YMF 1.06201
KT278952.1 Trichoderma afroharzianum strain LESF554 RNA polymerase II subunit 2 (rpb2) gene partial cds
PV130478.1:8-1077 Trichoderma asiaticum isolate be163 RNA polymerase II second largest subunit (RPB2) gene partial cds
PV130481.1:1-1068 Trichoderma asiaticum isolate be137 RNA polymerase II second largest subunit (RPB2) gene partial cds
PV130480.1:5-1074 Trichoderma asiaticum isolate be139 RNA polymerase II second largest subunit (RPB2) gene partial cds
FJ442686.1 Hypocrea lixii strain DIS 65V RNA polymerase II subunit (RPB2) gene partial cds
MG917685.1 Trichoderma harzianum strain T3 RNA polymerase II second largest subunit (RPB2) gene partial cds
KF134791.1 Trichoderma sp. HW-2014j strain S278 RNA polymerase II subunit 2 (rpb2) gene partial cds
KJ665273.1 Trichoderma guizhouense strain S628 RNA polymerase II subunit 2 (rpb2) gene partial cds
OP709971.1:10-1090 Trichoderma guizhouense Tr49
KJ665273.1:10-1084 Trichoderma guizhouense strain S628 RNA polymerase II subunit 2 (rpb2) gene partial cds
MN605870.1:1-1094 Trichoderma vermifimicola strain CGMCC 3.19850 RNA polymerase II second largest subunit (rpb2) gene partial cds
MN605871.1:1-1112 Trichoderma vermifimicola CGMCC 3.19694
MN605871.1 T. vermifimicola strain CGMCC 3.19694 rpb2 gene
MG873465.1 Trichoderma harzianum strain T8 RNA polymerase II second largest subunit (rpb2) gene partial cds
MG873464.1 Trichoderma harzianum strain T7 RNA polymerase II second largest subunit (rpb2) gene partial cds
OP832381.1:1-1114 Trichoderma notatum JZBQT1Z5
JQ901400.1 Trichoderma guizhouense strain HGUP0038 RNA polymerase II subunit (RPB2) gene partial cds
MG917683.1 Trichoderma harzianum strain T5 RNA polymerase II second largest subunit (RPB2) gene partial cds
FJ442710.1 Trichoderma simmonsii strain GJS 92-100 RNA polymerase II subunit (RPB2) gene partial cds
KJ665337.1:10-1084 Trichoderma simmonsii strain S7 RNA polymerase II subunit 2 (rpb2) gene partial cds
KT343765.1 Trichoderma sp. HW-2014w voucher HMAS 245000 RNA polymerase II subunit 2 (rpb2) gene partial cds
FJ442757.1 Trichoderma simmonsii strain GJS 91-138 RNA polymerase II subunit (RPB2) gene partial cds
KJ665337.1 Trichoderma simmonsii strain S7 RNA polymerase II subunit 2 (rpb2) gene partial cds
KY688003.1 Trichoderma bannaense voucher HMAS:248865 RNA polymerase subunit II (RPB2) gene partial cds
KY687979.1 Trichoderma bannaense voucher HMAS:248840 RNA polymerase subunit II (RPB2) gene partial cds
KY687979.1:20-1086 Trichoderma bannaense voucher HMAS:248840 RNA polymerase subunit II (RPB2) gene partial cds
MN450663.1 Trichoderma sp. strain mms1255 RNA polymerase subunit II (RPB2) gene partial cds
KX632575.1:20-1109 Trichoderma alni strain T24 RNA polymerase II subunit (RPB2) gene partial cds
KX632575.1 Trichoderma alni strain T24 RNA polymerase II subunit (RPB2) gene partial cds
FJ442779.1 Hypocrea lixii strain GJS 04-71 RNA polymerase II subunit (RPB2) gene partial cds
XM 024924757.1:1305-2394 Trichoderma harzianum CBS 226.95 rpb2 gene
KX632534.1 Trichoderma harzianum strain T2 RNA polymerase II subunit (RPB2) gene partial cds
KX632533.1 Trichoderma harzianum strain T1 RNA polymerase II subunit (RPB2) gene partial cds
MH647801.1 Trichoderma harzianum isolate HZA11 RNA polymerase subunit II (RPB2) gene partial cds
FJ442708.1 Trichoderma harzianum strain GJS 05-107 RNA polymerase II subunit (RPB2) gene partial cds
KX632543.1 Trichoderma harzianum strain T11 RNA polymerase II subunit (RPB2) gene partial cds
MT118249.1 Trichoderma harzianum isolate Thaum14 RNA polymerase II subunit (RPB2) gene partial cds
KX632561.1 Trichoderma harzianum strain T35 RNA polymerase II subunit (RPB2) gene partial cds
KT278951.1 Trichoderma harzianum strain LESF121 RNA polymerase II subunit 2 (rpb2) gene partial cds
EU498358.1 Trichoderma brunneoviride strain CBS120928 RNA polymerase subunit II (rpb2) gene partial cds
ON649976.1:1-1113 Trichoderma pleuroticola isolate JZBQT3Z1 RNA polymerase II subunit 2 (rpb2) gene partial cds
PQ112259.1:1-1111 Trichoderma pleuroticola strain MSU FS-06018 RNA polymerase II second largest subunit (RPB2) gene partial cds
MF371205.1 Trichoderma sp. strain TC139 RNA polymerase subunit II (rpb2) gene partial cds
MF371203.1 Trichoderma sp. strain TC62 RNA polymerase subunit II (rpb2) gene partial cds
OQ026385.1:1-1063 Trichoderma pleuroticola GP Bl-6
ON649968.1:1-1113 Trichoderma miyunense isolate JZBQF5 RNA polymerase II subunit 2 (rpb2) gene partial cds
ON649969.1:1-1113 Trichoderma miyunense isolate JZBQF7 RNA polymerase II subunit 2 (rpb2) gene partial cds
ON649970.1:1-1113 Trichoderma miyunense isolate JZBQF9 RNA polymerase II subunit 2 (rpb2) gene partial cds
KY687988.1 Trichoderma solum voucher HMAS:248849 RNA polymerase subunit II (RPB2) gene partial cds
KY687986.1 Trichoderma solum voucher HMAS:248847 RNA polymerase subunit II (RPB2) gene partial cds
KY687987.1 Trichoderma solum voucher HMAS:248848 RNA polymerase subunit II (RPB2) gene partial cds
KX632574.1:20-1109 Trichoderma alni strain T16 RNA polymerase II subunit (RPB2) gene partial cds
KX632576.1:20-1109 Trichoderma alni strain T28 RNA polymerase II subunit (RPB2) gene partial cds
KJ665244.1:10-1084 Trichoderma christiani culture CBS:132572 strain S442 voucher WU:33379 RNA polymerase II subunit 2 (rpb2) gene partial cds
KJ665245.1:10-1083 Trichoderma christiani S93
KF730007.1 Trichoderma rufobrunneum isolate 8155 RNA polymerase II second largest subunit (RPB2) gene partial cds
KJ665332.1 Trichoderma priscilae strain S129 RNA polymerase II subunit 2 (rpb2) gene partial cds
KJ665332.1:10-1084 Trichoderma priscilae S129
KT343764.1 Trichoderma priscilae voucher HMAS 245002 RNA polymerase II subunit 2 (rpb2) gene partial cds
KX026961.1 Trichoderma sp. 2 WYZ-2016 strain HMAS 273787 RNA polymerase II second largest subunit (rpb2) gene partial cds
KF134789.1:1-1113 Trichoderma compactum strain CBS 121218 RNA polymerase II subunit 2 (rpb2) gene partial cds
KF134789.1 Trichoderma compactum strain CBS 121218 RNA polymerase II subunit 2 (rpb2) gene partial cds
KY688010.1 Trichoderma ingratum voucher HMAS:248873 RNA polymerase subunit II (RPB2) gene partial cds
KY687966.1 Trichoderma ingratum voucher HMAS:248827 RNA polymerase subunit II (RPB2) gene partial cds
KY687964.1 Trichoderma ingratum voucher HMAS:248824 RNA polymerase subunit II (RPB2) gene partial cds
KY687965.1 Trichoderma ingratum voucher HMAS:248826 RNA polymerase subunit II (RPB2) gene partial cds
KF134794.1 Trichoderma velutinum strain C.P.K. 298 RNA polymerase II subunit 2 (rpb2) gene partial cds
KF134788.1 Trichoderma cerinum strain S357 RNA polymerase II subunit 2 (rpb2) gene partial cds
MT587315.1:6-1117 Trichoderma lixii strain TLiC8 RNA polymerase II second largest subunit (RPB2) gene partial cds
OP832378.1:1-1112 Trichoderma lentinulae isolate JZBQT0Z1 RNA polymerase II subunit 2 (rpb2) gene partial cds
MN605877.1:1-1112 Trichoderma lentinulae strain CGMCC 3.19700 RNA polymerase II second largest subunit (rpb2) gene partial cds
MN605868.1:1-1112 Trichoderma lentinulae strain CGMCC 3.19848 RNA polymerase II second largest subunit (rpb2) gene partial cds
MN605874.1:1-1112 Trichoderma xixiacum strain CGMCC 3.19697 RNA polymerase II second largest subunit (rpb2) gene partial cds
MN605867.1:1-1110 Trichoderma lentinulae strain CGMCC 3.19847 RNA polymerase II second largest subunit (rpb2) gene partial cds
KP171658.1:1-655 Rhizoctonia endophytica strain UAOM 138188 RNA polymerase II second largest subunit (Rpb2) gene partial cds
0.10
