# Supplementary material for: Exploring the Bioactive Secondary Metabolites of Two Argentine Trichoderma afroharzianum Strains
Source: J Fungi (Basel). 2025 Jun 17;11(6):457. doi: 10.3390/jof11060457 (PMC12194781; doi:10.3390/jof11060457)
Supplement: Supplementary file 1 [file jof-11-00457-s001.zip › Supplementary File.pdf]

Table S1: Percentage of identity for the *rpb2* gene of strains 10BR1 and UEPA AR12 compared to other *Trichoderma* strains based on BLASTn analysis.

Species	Strain	Similarity (%)	Total Score	E-value	GenBank accession number
		10BR1			
T. afroharzianum	Tr96	99.82%	2045	0.0	OP374181.1
T. afroharzianum	Tr84A	99.82%	2045	0.0	OP374178.1
T. afroharzianum	Tr129	99.82%	2045	0.0	OP374183.1
T. afroharzianum	Tr138B	99.82%	2045	0.0	OP374185.1
T. afroharzianum	Tr132	99.82%	2045	0.0	OP374184.1
T. afroharzianum	Tr28	99.82%	2045	0.0	OP374175.1
T. afroharzianum	Tr90A	99.82%	2045	0.0	OP374179.1
T. afroharzianum	Tr153	99.82%	2045	0.0	OP374186.1
T. afroharzianum	Tr95	99.82%	2045	0.0	OP374180.1
T. afroharzianum	Tr48	99.82%	2045	0.0	OP374176.1
		UEPA AR12			
T. afroharzianum	Tri-1	100.00%	2049	0.0	OP102132.1
T. afroharzianum	Tr84A	99.82%	2037	0.0	OP374178.1
T. afroharzianum	Tr129	99.82%	2037	0.0	OP374183.1
T. afroharzianum	Tr96	99.82%	2037	0.0	OP374181.1
T. afroharzianum	Tr138B	99.82%	2037	0.0	OP374185.1
T. afroharzianum	Tr132	99.82%	2037	0.0	OP374184.1
T. afroharzianum	Tr28	99.82%	2037	0.0	OP374175.1
T. afroharzianum	Tr90A	99.82%	2037	0.0	OP374179.1
T. afroharzianum	Tr153	99.82%	2037	0.0	OP374186.1
T. afroharzianum	Tr95	99.82%	2037	0.0	OP374180.1

Only the top 10 matches with highest percent ID as well as those with the type strains (T) are shown.

Table S2: Selectivity index (SI = IC_{50} / MIC) of *T. afroharzianum* extracts CSE10BR1 and CSEAR12 against microbial strains based on different human cell lines.

**Strain**	**MIC (µg/mL) (CSE10BR1 / CSEAR12)**	**Selectivity index - IC_{50} / MIC (CSE10BR1 / CSEAR12)**			
		**HaCaT (IC₅₀ 368.7 / 202.5 µg/mL)**	**HEK293 (IC₅₀ 437.8 / 217.5 µg/mL)**	**Vero (IC₅₀ 593.7 / 234.3 µg/mL)**	**Huh7 (IC₅₀ 602.1 / 182.5 µg/mL)**
***S. aureus* (ATCC 25923)**	31.25 / 15.6	**11.8 / 13.0**	**14.0 / 13.9**	**19.0 / 15.0**	**19.3 / 11.7**
***E. faecalis* (ATCC 29212)**	250 / 125	1.5 / 1.6	1.8 / 1.7	2.4 / 1.9	2.4 / 1.5
***E. coli* (ATCC 25922 & 35218)**	500 / 500	0.74 / 0.41	0.88 / 0.43	1.19 / 0.47	1.20 / 0.36
***S.* Typhimurium (ATCC 14028s)**	250 / 250	1.47 / 0.81	1.75 / 0.87	2.37 / 0.94	2.41 / 0.73
***C. albicans* (ATCC 10231)**	1000 / 1000	0.37 / 0.20	0.44 / 0.22	0.59 / 0.23	0.60 / 0.18
***C. parapsilosis* (ATCC 22019)**	>1000 / 1000	ND / 0.20	ND / 0.22	ND / 0.23	ND / 0.18
***C. tropicalis* (ATCC 200956)**	1000 / 500	0.37 / 0.41	0.44 / 0.44	0.59 / 0.47	0.60 / 0.36
***C. krusei* (ATCC 6258)**	500 / 250	0.74 / 0.81	0.88 / 0.87	1.19 / 0.94	1.20 / 0.73
***C. glabrata* (ATCC 2950)**	1000 / >1000	0.37 / ND	0.44 / ND	0.59 / ND	0.60 / ND
***C. albicans* (CCC 191-13)**	1000 / 1000	0.37 / 0.20	0.44 / 0.22	0.59 / 0.23	0.60 / 0.18
***C. albicans* (CCC 132-15)**	>1000 / 1000	ND / 0.20	ND / 0.22	ND / 0.23	ND / 0.18

Notes: The SI was calculated as the ratio of the IC_{50} (µg/mL) in each mammalian cell line to the MIC (µg/mL) against the corresponding microorganism (SI = IC_{50} / MIC). SI > 10: Indicates high selectivity, meaning the extract inhibits the microorganism at concentrations well below those toxic to mammalian cells, suggesting a favorable safety profile; SI between 1 and 10: Indicates moderate selectivity, where antimicrobial and cytotoxic concentrations are closer, requiring cautious interpretation regarding safety; SI < 1: Indicates low selectivity, with antimicrobial activity occurring at concentrations similar to or lower than toxic levels, suggesting potential safety concerns. Values in bold indicate promising selectivity (SI > 10). ND = Not determined.

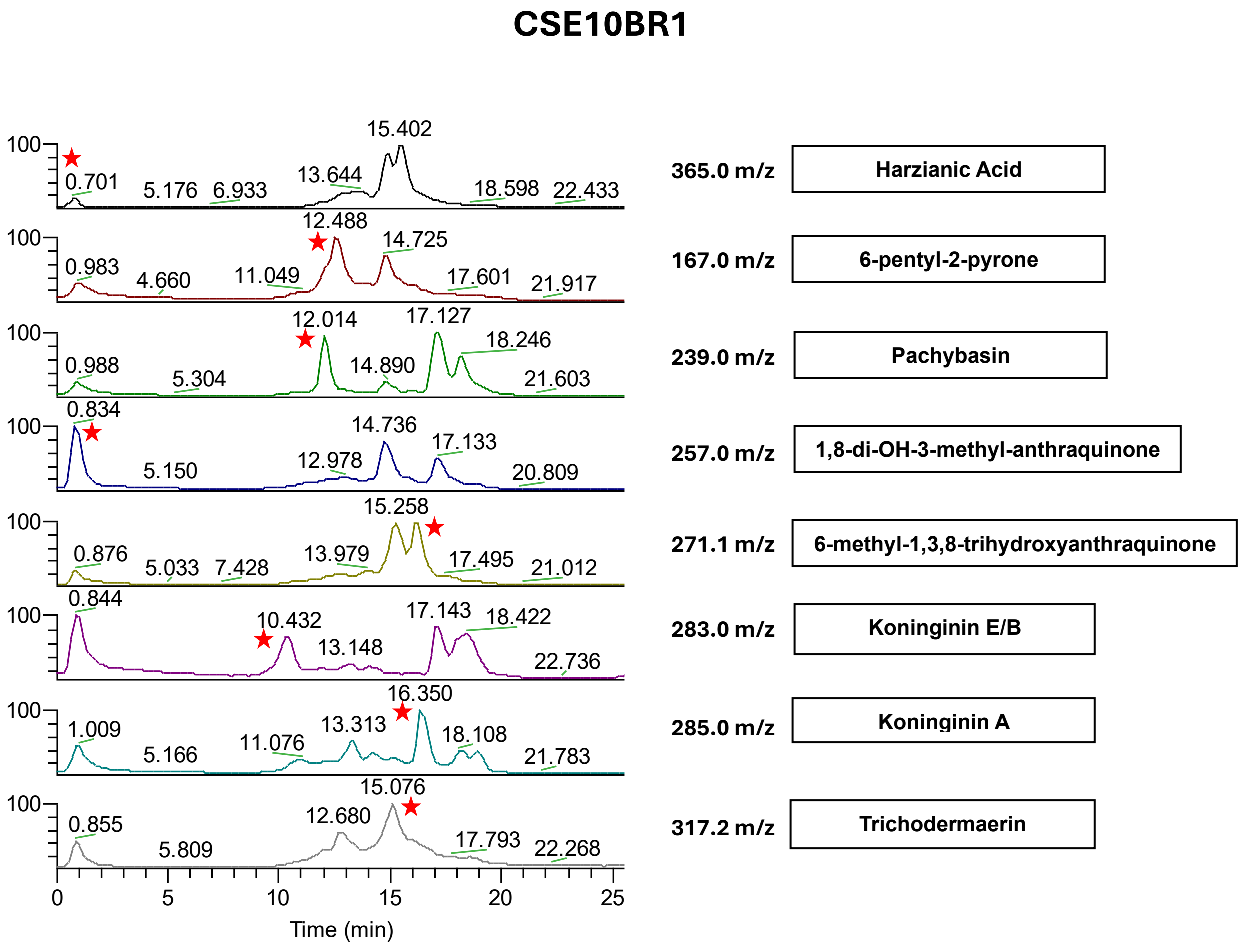

Figure S2. HPLC-MS chromatograms of the main metabolites detected in the culture supernatant extract of *T. afroharzianum* strain 10BR1 (CSE10BR1).

CSEAR12

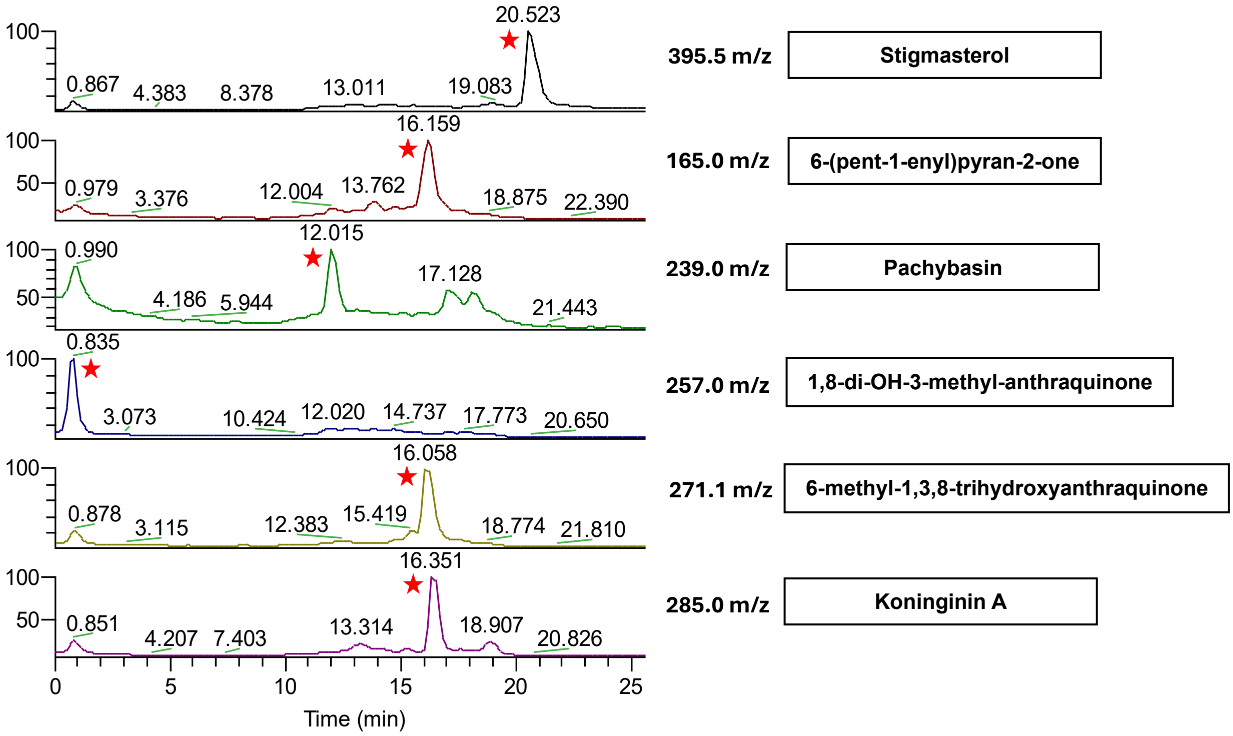

Figure S3: HPLC-MS chromatograms of the main metabolites detected in the culture supernatant extract of *T. afroharzianum* strain UEPA AR12 (CSEAR12).

Table S3: Putative biosynthetic pathways for metabolites identified by HPLC-MS, based on FUNGISMASH genome analysis.

Compound	Putative Biosynthetic Pathway(s)
6-(pent-1-enyl)pyran-2-one	T1PKS
6-pentyl-2-pyrone	T1PKS
1-hydroxy-3-methyl-anthraquinone (Pachybasin)	T1PKS, NRPS
1,8-dihydroxy-3-methyl-anthraquinone	T1PKS
6-methyl-1,3,8-trihydroxyanthraquinone	T1PKS
Harzianopyridone	NRPS, T1PKS
Koninginins (A, B/E)	PKS-NRPS hybrid
Trichodermaerin	NRPS
Harzianic acid	T1PKS, NRPS
Saturnispol C	PKS-NRPS hybrid
Stigmasterol	Terpene
Trichotetronine	Fungal-RiPP-like, T1PKS
Atrichodermone C	T1PKS
Methylcordysinin A	NRPS
Ergosta-7,22-dien-3-ol	Terpene
β-sitosterol	Terpene
3,5,9-trihydroxyergosta-7,22-dien-6-one	Terpene
Aspereline A	NRPS
Aspereline E	NRPS
Aspereline H	NRPS

Note: T1PKS refers to Type I Polyketide Synthase, a key enzyme involved in the synthesis of polyketides. NRPS stands for Non-Ribosomal Peptide Synthase, responsible for the production of non-ribosomal peptides. The NRPS–PKS hybrid represents a cluster that combines both NRPS and PKS modules for the synthesis of hybrid compounds. Fungal-RiPP-like refers to ribosomally synthesized peptides that are enzymatically modified, a characteristic of fungal secondary metabolites. Lastly, Terpene (mevalonate pathway) refers to compounds derived from the mevalonate pathway, a common route for terpene biosynthesis in fungi.

Table S4: Genomic regions associated with secondary metabolite biosynthesis in *T. afroharzianum* 10BR1 according to FUNGISMASH.

Region	Type	From	To	Most similar known cluster		Similarity
Region 29.1	Terpene	57,318	88,588			
Region 29.2	NRPS-like	125,027	188,878	Choline	NRP	100%
Region 36.1	T1PKS	150,151	213,097			
Region 38.1	T1PKS	1	57,586	Depudecin	Polyketide:Iterative type I polyketide	33%
Region 39.1	T1PKS, NRPS, betalactona	19,911	91,918	Harzianopyridone	NRP+Polyketide:Iterative type I	60%
Region 40.1	T1PKS	1	63,493	Clavaric acid	Terpene	100%
Region 43.1	NRPS	1	61,591			
Region 72.1	T1PKS	1	60,656			
Region 75.1	T1PKS	165,428	230,111	Decumbenone A / Calbistrin A / Calbistrin C / Decumbenone B / Decumbenone C / Dioic acid moiety	Polyketide	15%
Region 75.2	Fungal-RiPP-like	502,973	593,522			
Region 75.3	NRPS, T1PKS	705,692	844,490	Harziphilone / T22azaphilone / Isoharziphilone-1 / Isoharziphilone-2 / Compound 4 / Compound 1	Polyketide	90%
Region 79.1	T1PKS	356,192	417,422			
Region 81.1	NRPS, T1PKS	1	108,622			
Region 94.1	NRPS	47,518	114,052	Peramina / Intermediary 1 / Intermediary 2	NRP	100%
Region 103.1	NRPS-like	248,711	299,818			
Region 103.2	NRPS	333,661	413,305			
Region 105.1	NRPS-like	1	56,546			
Region 106.1	NRPS	39,975	148,485			
Region 107.1	Fungal-RiPP-like	206,591	297,381			
Region 110.1	Terpene	24,297	55,355			
Region 116.1	T1PKS	370,916	438,623			
Region 119.1	T1PKS	102,192	168,887	Aurofusarin	Polyketide	18%
Region 120.1	Terpene	1	22,392	Squalestatin S1	Terpene	40%
Region 135.1	NRPS-like	80,406	144,173			
Region 144.1	Fungal-RiPP-like	101,395	161,597			
Region 148.1	T1PKS	209,669	279,398			
Region 170.1	NRPS, T1PKS	47,231	150,603			
Region 171.1	T1PKS	76,007	141,370			

Region 172.1	NRPS	225,742	288,707			
Region 173.1	T1PKS	78,287	146,878			
Region 175.1	NRPS-like	31,541	93,392			
Region 177.1	T1PKS, NRPS	386	73,065			
Region 177.2	Terpene	96,768	128,351	Trichobrasilenol / Xylarenic acid B / Brasilane A / Brasilane F / Brasilane E / Brasilane D	Terpene	60%
Region 177.3	NRPS	153,870	222,408			
Region 193.1	T1PKS, NRPS	1	119,337			
Region 194.1	T1PKS	1	40,264			
Region 194.2	Terpene	324,553	356,080			
Region 201.1	Fungal-RiPP-like	1	49,770			
Region 201.2	T1PKS	205,768	256,021	Cryptosporioptide B / Cryptosporioptide A / Cryptosporioptide C	Polyketide:Iterative type I polyketide	23%
Region 202.1	NRPS	22,917	100,350			
Region 205.1	T1PKS	26,851	102,969	Tricholignan A	Polyketide:Iterative type I polyketide	77%
Region 207.1	T1PKS	28,712	94,186			
Region 220.1	NRPS-like	1	42,253			
Region 226.1	NRPS, T1PKS	59,415	130,498			
Region 226.2	T1PKS	160,222	226,705			
Region 246.1	T1PKS	1	52,598	Dichlorodiaportin	Polyketide	50%
Region 269.1	Fungal-RiPP-like	10,473	61,137			
Region 271.1	NRPS	47,853	84,286	Metachelin C/metachelin A/metachelin A-CE/metachelin B/dimerumic acid 11-mannoside/dimerumic acid	NRP	25%
Region 299.1	NRPS, T1PKS	51,227	124,754	Phyllostictine A / Phyllostictine B	NRP + Polyketide	40%
Region 316.1	Terpene	1	20,697			
Region 321.1	T1PKS	1	46,146	Trichoxide	Polyketide	75%

Table S5: Genomic regions associated with secondary metabolite biosynthesis in *T. afroharzianum* UEPA AR12 according to FUNGISMASH.

Region	Type	From	To	Most similar known cluster		Similarity
Region 1.1	T1PKS	284,440	352,038	Trichoxide	Polyketide	100%
Region 23.1	NRPS-like	22,942	86,020			
Region 30.1	Terpene	1	30,181			
Region 35.1	Terpene	80,503	111,443			
Region 49.1	NRPS-like	300	56,506			
Region 49.2	NRPS, T1PKS	84,011	206,821			
Region 52.1	T1PKS	1	40,256			
Region 59.1	T1PKS	348,276	416,870			
Region 63.1	T1PKS, Terpene	231,131	299,750	Sordarin	Polyketide	35%
Region 78.1	Terpene	40,906	72,285			
Region 79.1	T1PKS	1	45,894	Cryptosporioptide B / Cryptosporioptide A / Cryptosporioptide C	Polyketide: Iterative type I polyketide	23%
Region 94.1	Fungal-RiPP-like	59,130	149,920			
Region 95.1	NRPS	20,454	128,964			
Region 101.1	Terpene	19,484	51,084	Squalestatin S1	Terpene	40%
Region 102.1	NRPS	47,786	113,293	Metachelin C / Metachelin A / Metachelin A-CE / Metachelin B / Dimerumic acid 11-mannoside / Dimerumic acid	NRP	100%
Region 109.1	NRPS	1	53,065			
Region 114.1	T1PKS	97,968	167,885			
Region 121.1	NRPS, T1PKS, betalactone	97,267	157,642	Harzianopyridone	NRP + Polyketide:Iterative type I polyketide	60%
Region 127.1	T1PKS, NRPS	92,616	166,143	Phyllostictine A / Phyllostictine B	NRP + Polyketide	40%
Region 129.1	T1PKS	105,955	171,490			
Region 138.1	T1PKS	133,775	199,240	Aurofusarin	Polyketide	27%
Region 146.1	T1PKS	8,237	73,597			
Region 147.1	NRPS	564,147	625,228			
Region 158.1	NRPS	1	81,325			
Region 167.1	T1PKS, NRPS	1	106,085			
Region 178.1	T1PKS	592	68,459	Depudecin	Polyketide: Iterative type I polyketide	33%
Region 182.1	NRPS-like, NRPS, T1PKS	71	111,215			
Region 193.1	Fungal-RiPP-like, NRPS-like	84,595	189,227	Choline	NRP	100%

Region 193.2	Terpene	226,359	257,627			
Region 198.1	Fungal-RiPP-like	9,692	103,151			
Region 202.1	NRPS	11,074	76,803			
Region 213.1	Fungal-RiPP-like	1,648	67,705			
Region 218.1	Terpene	33,131	65,134	Trichobrasilenol / Xylarenic acid B / Brasilane A / Brasilane F / Brasilane E / Brasilane D	Terpene	60%
Region 218.2	T1PKS,NRPS	88,812	154,329			
Region 237.1	T1PKS	43,521	125,539	Dichlorodiaporthin	Polyketide	50%
Region 251.1	Fungal-RiPP-like	89,140	179,689			
Region 273.1	Fungal-RiPP-like	28,189	118,688			
Region 277.1	NRPS-like	71,995	136,930			
Region 280.1	T1PKS,NRPS	18,942	157,961	Harziphilone / T22azaphilone / Isoharziphilone-1 / Isoharziphilone-2 / Compound 4 / Compound 1	Polyketide	80%
Region 296.1	T1PKS	142,021	220,537	Tricholignan A	Polyketide:Iterative type I polyketide	77%
Region 311.1	NRPS	11,106	45,125			
Region 311.2	T1PKS,NRPS	134,689	205,772			
Region 325.1	T1PKS	58,012	105,010			
Region 342.1	NRPS-like	1	48,348			
Region 353.1	T1PKS	1	11,523			
Region 379.1	Terpene	2,362	25,544	Copalyl diphosphate	Terpene	28%
Region 384.1	T1PKS	1	51,073	Decumbenone A / Calbistrin A / Calbistrin C / Decumbenone B / Decumbenone C / Dioic acid moiety	Polyketide	15%
Region 396.1	NRPS	1	45,118			
Region 460.1	T1PKS	1	29,523			
